# Translatome proteomics identifies autophagy as a resistance mechanism to on-target FLT3 inhibitors in acute myeloid leukemia

**DOI:** 10.1038/s41375-022-01678-y

**Published:** 2022-08-23

**Authors:** Sebastian E. Koschade, Kevin Klann, Shabnam Shaid, Binje Vick, Jan A. Stratmann, Marlyn Thölken, Laura M. Meyer, The Duy Nguyen, Julia Campe, Laura M. Moser, Susanna Hock, Fatima Baker, Christian T. Meyer, Frank Wempe, Hubert Serve, Evelyn Ullrich, Irmela Jeremias, Christian Münch, Christian H. Brandts

**Affiliations:** 1 Department of Medicine, Hematology/Oncology, University Hospital, Goethe University Frankfurt, Frankfurt, Germany; 2 Institute of Biochemistry II, Faculty of Medicine, Goethe University Frankfurt, Frankfurt, Germany; 3 German Cancer Consortium (DKTK), Heidelberg, Germany; 4 German Cancer Research Center (DKFZ), Heidelberg, Germany; 5 Frankfurt Cancer Institute, Goethe University Frankfurt, Frankfurt, Germany; 6 University Cancer Center Frankfurt (UCT), University Hospital, Goethe University Frankfurt, Frankfurt, Germany; 7 Helmholtz Zentrum München, German Research Center for Environmental Health, Research Unit Apoptosis in Hematopoietic Stem Cells, Munich, Germany; 8 Department for Children and Adolescents, Experimental Immunology, University Hospital, Goethe University Frankfurt, Frankfurt, Germany; 9 Department for Children and Adolescents, Division for Stem Cell Transplantation, Immunology and Intensive Care Medicine, University Hospital, Goethe University Frankfurt, Frankfurt, Germany; 10 Vanderbilt University, Center for Cancer Systems Biology at Vanderbilt, Nashville, TN USA; 11 Department of Pediatrics, Dr. von Hauner Children’s Hospital, University Hospital, LMU Munich, Munich, Germany; 12 Cardio-Pulmonary Institute, Frankfurt, Germany

**Keywords:** Acute myeloid leukaemia, Cancer therapeutic resistance, Preclinical research

## Abstract

Internal tandem duplications (ITD) in the receptor tyrosine kinase FLT3 occur in 25 % of acute myeloid leukemia (AML) patients, drive leukemia progression and confer a poor prognosis. Primary resistance to FLT3 kinase inhibitors (FLT3i) quizartinib, crenolanib and gilteritinib is a frequent clinical challenge and occurs in the absence of identifiable genetic causes. This suggests that adaptive cellular mechanisms mediate primary resistance to on-target FLT3i therapy. Here, we systematically investigated acute cellular responses to on-target therapy with multiple FLT3i in FLT3-ITD + AML using recently developed functional translatome proteomics (measuring changes in the nascent proteome) with phosphoproteomics. This pinpointed AKT-mTORC1-ULK1-dependent autophagy as a dominant resistance mechanism to on-target FLT3i therapy. FLT3i induced autophagy in a concentration- and time-dependent manner specifically in FLT3-ITD + cells in vitro and in primary human AML cells ex vivo. Pharmacological or genetic inhibition of autophagy increased the sensitivity to FLT3-targeted therapy in cell lines, patient-derived xenografts and primary AML cells ex vivo. In mice xenografted with FLT3-ITD + AML cells, co-treatment with oral FLT3 and autophagy inhibitors synergistically impaired leukemia progression and extended overall survival. Our findings identify a molecular mechanism responsible for primary FLT3i treatment resistance and demonstrate the pre-clinical efficacy of a rational combination treatment strategy targeting both FLT3 and autophagy induction.

## Introduction

Acute myeloid leukemia (AML) is an aggressive malignancy of the hematopoietic system and the most frequent form of acute leukemias in adults [1]. Activating internal tandem duplications (ITD) in the receptor tyrosine kinase FLT3 occur in 25% of AML patients. This recurrent somatic mutation promotes AML cell survival and proliferation [2, 3], confers a poor prognosis [4] and is an attractive target for treatment. Physiologic expression of FLT3 is mostly limited to transient hematopoietic progenitor cells [5], suggesting a large therapeutic index.

Quizartinib [6], crenolanib [7] and gilteritinib [8] are 2nd generation FLT3 inhibitors (FLT3i) in phase III trials or clinical use for the targeted treatment of FLT3-ITD + AML. Their clinical benefit is challenged by intrinsic resistance phenotypes, leading to poor primary response and the rapid development of acquired resistance in the majority of patients [9–11]. A full understanding of cellular resistance factors contributing to poor primary responses in the absence of identifiable genetic causes is lacking. Several prior studies suggested that adaptive mechanisms may mediate primary resistance to FLT3i [12–14].

We addressed this issue from a new perspective by investigating nascent proteome dynamics and phospho-signaling upon FLT3i to identify non-genetic mechanisms of primary treatment resistance. Regulation at the level of protein translation is recognized as the predominant mode of gene expression control [15]. Translation is an essential adaptive mechanism to reshape cellular proteomes in response to perturbations to maintain homeostasis and cell survival [15]. Translation dynamics of human cancers in response to therapy have not yet been explored quantitatively on a systems scale using perturbation-free proteomic measurement techniques [16].

Our systems biology approach revealed a specific translation and phospho-signaling response pinpointing autophagy as a dominant primary resistance mechanism against on-target anti-FLT3 therapy. (Macro-)Autophagy is an adaptive intracellular pathway that is activated to maintain cellular homeostasis by targeting cytosolic constituents to the lysosome for degradation and recycling [17]. Autophagy can contribute to treatment resistance in various cancers, but has also been shown to mediate anti-tumor effects, and its specific functions are highly context-dependent [17]. Autophagy appears to mediates both pro-tumor [18, 19] and antitumor [20, 21] effects relevant to FLT3-ITD + AML cell proliferation under basal conditions, and its function in treatment resistance to FLT3 therapy is unclear. Using multiple genetic and pharmacologic models, we show that on-target FLT3 therapy induces a cell-autonomous autophagy response that can be therapeutically exploited to significantly enhance the antileukemic activity of FLT3-targeted therapy. Our results open new perspectives for the study of translation dynamics and therapy resistance in cancer and suggest the clinical evaluation of combined FLT3 and autophagy inhibition to overcome primary treatment resistance against FLT3-targeted therapy and improve its efficacy.

## Methods

### Cell lines and compounds

Cells were cultured at 37 °C with 5% CO_2_ in a humidified Heracell 150i incubator (Thermo Fisher Scientific) and regularly confirmed to be mycoplasma-free [22]. Cells were used at low passage number for a maximum of 3 months. 32Dcl3 (32D) and AML cells MV4-11, MOLM-14, HEL-276, HL-60, OCI-AML-3, THP-1 (Leibniz-Institut DSMZ) were cultured in RPMI1640 medium (Gibco, Thermo Fisher Scientific) supplemented with 10% FBS (Sigma-Aldrich) and 1% penicillin/streptomycin (Thermo Fisher Scientific) and 1% L-glutamin (Sigma-Aldrich). 32Dcl3 cells were additionally supplemented with 1 ng/ml murine IL-3 (PeproTech). Platinum-E (Cell Biolabs, Inc.) and Lenti-X 293 T cells (Clontech, Takara Bio) were cultured in DMEM (Gibco, Thermo Fisher Scientific) supplemented with 10% FBS, 1% penicillin/streptomycin and 1% L-glutamin. Cells were authenticated by suppliers. Drugs (Selleckchem) were dissolved in DMSO, aliquoted for single use and stored at −80 °C.

### Translatome proteomics and phospho-proteomics

For translatome proteomics, cells were resuspended in pre-warmed RPMI1640 SILAC medium (Gibco) containing 100 µg/mL Arg10, 100 µg/mL Lys8 (Cambridge Isotope Laboratories), 10% FBS and 10 nM of quizartinib, crenolanib, gilteritinib or DMSO. Primary cells were resuspended in pre-warmed IMDM for SILAC (Gibco, Thermo Fisher Scientific) supplemented with 84 µg/mL Arg10, 146 µg/mL Lys8, 10% (v/v) BIT9500 (STEMCELL Technologies), 10 µg/ml human LDL (Low Density Lipoprotein, STEMCELL Technologies), 55 µM 2-mercaptoethanol (Gibco, Thermo Fisher Scientific), FLT3 ligand, IL-3, SCF (human, 10 ng/ml each, all PeptroTech) and 10 nM of quizartinib, crenolanib, gilteritinib or DMSO. After 6 h cells were collected (300 *g*, 5 min) into 1.5 mL Protein LoBind tubes (Eppendorf), washed 3× (PBS) and then lysed with fresh lysis buffer (2% w/v sodium deoxycholate, 100 mM Tris-HCl pH 8, 2.5 mM TCEP, 10 mM chloracetamide, 95 °C). Samples were incubated for 5 min at 95 °C and then sonicated with Sonic Vibra Cell at 1 s ON/1 s OFF pulse for 30 s at 30% amplitude. For phospho-proteomics, cells were treated in non-SILAC medium and lysis buffer additionally contained 1× PhosSTOP (Roche, Switzerland) phosphatase inhibitor.

### Autophagy assays using stable flux reporter cell lines

Cells were gated by forward and side scatter, followed by doublette exclusion. Fluorescence eGFP (B-530/30), mCherry (G-610/20), or RFP (G-582/15) signal intensities were measured. Autophagic flux was quantified by ratiometric single-cell values (eGFP/mCherry, eGFP/RFP) and aggregated per sample by the arithmetic mean.

### Autophagy assay by confocal microscopy imaging

Cells were deposited on cytospins, fixed and permeabilized in methanol (−20 °C, 15 min), blocked (BlockAid; Thermo Fisher Scientific), and rabbit anti-LC3A/B mAb Alexa Fluor 647 Conjugate (13394, 1:50, Cell Signaling Technology) in PBS + 1% BSA + 0.3% Triton X-100 was applied overnight (4 °C), followed by 300 nM DAPI (AnaSpec, 5 min). After mounting (Prolong Diamond Antifade, Thermo Fisher Scientific) and drying overnight (4 °C), images were acquired using a Leica TCS SP5 II confocal laser scanning microscope (HCX PL APO CS 63×/1.4N oil immersion objective) and LAS-AF lite 2.0 (Leica Microsystems).

### In vivo treatment trial

MV4-11-eGFP-LC3B-mCherry cells were transduced with VSV-G-pseudotyped lentiviral vector encoding firefly luciferase and blasticidin resistance (linked via a P2A peptide) and selected by blasticidin (8 µg/ml). Six–eight week old male and female NSG (non-obese diabetic (NOD)/severe combined immunodeficient (SCID)/Il2rg−/−) mice were injected with 1 × 10^6^ cells into the dorsal tail vein. Two weeks later, animals were randomized and treated daily until death by oral gavage application at 6 µl/g body weight. Drugs (Selleckchem) were suspended in 0.5% (w/v) methylcellulose, aliquoted and stored at −80 °C. Disease development was monitored by longitudinal in vivo imaging with an IVIS Lumina II (Perkin Elmer). Bioluminescence was measured at serial exposure times (10 s–4 min) from the dorsal and ventral view 15 min after subcutaneous injection of 100 µl D-luciferin (1.5 mg/ml; Promega). All applicable guidelines for the housing, care and use of animals were followed. Moribund mice were sacrificed. Animal experiments were approved by the responsible government oversight committee (Regierungspräsidium Darmstadt, Dezernat V54, ref. FK/1112). Survival analyses were performed using Kaplan-Meier method and Cox robust regression (clustered by transplantation cohort) implemented in R’s survival 3.1.12 package [23]. All animals assigned to treatment groups were included in the analysis.

### Patient-derived primary AML cells

Between 2015 and 2019, bone marrow (BM) aspiration samples [10] or leukapheresates [1] from 11 untreated patients with newly diagnosed AML according to WHO criteria [24] were obtained after written informed consent in accordance with the Declaration of Helsinki. The ethics review board of the Goethe University (Frankfurt, Germany) approved the biomaterial usage (ref. SHN-07-2017). AML cells were isolated from heparinized BM aspirates or leukapheresate by Ficoll-Hypaque density-gradient centrifugation and stored in liquid nitrogen (biobank of the University Cancer Center Frankfurt, UCT Frankfurt, German Biobank Alliance). Samples were thawed in a water bath at 37 °C, washed 3× (250 *g*, 5 min) with IMDM medium (Gibco, Thermo Fisher Scientific) containing 10 µg/ml DNase I (Millipore, Sigma-Aldrich) and 5% FBS and resuspended in complete serum-free growth medium (cSFM) composed of IMDM without phenol red (Gibco, Thermo Fisher Scientific), 20% (v/v) BIT9500 (STEMCELL Technologies), 10 µg/ml human LDL (Low Density Lipoprotein, STEMCELL Technologies), 55 µM 2-mercaptoethanol (Gibco, Thermo Fisher Scientific), FLT3 ligand, IL-3, and SCF (human, 10 ng/ml each, all PeptroTech).

### Cyto-ID autophagy assay

Cells were seeded at a density of 0.5 × 10^6^ cells/ml in cSFM and treated as indicated. After 18 h at 37 °C/5% CO_2_, 60 µM chloroquine (Bayer) or H_2_O vehicle were added for another 6 h. Cells were stained with Cyto-ID Green 2.0 (Enzo) per the manufacturer’s recommendations, with surface antibodies for 30 min at 4° (PE-Cy7 mouse anti-human CD3 (BD, #557749, SP34-2), PE-Cy7 mouse anti-human CD19 (BD, #560728, HIB19), APC mouse anti-human CD33 (BD, #551378, WM53), BV421 mouse anti-human CD34 (BD, #562577, 581), APC-H7 mouse anti-human CD38 (BD, #656646, HB7), BV711 mouse anti-human CD45 (BD, #564358, HI30) and 7-AAD/Annexin V staining solution (140 mM NaCl, 4 mM KCl, 0.75 mM MgCl_2_, 10 mM HEPES, 85 mM CaCl_2_ in H_2_O [25]) with 1 µl 7-AAD (BD, #559925)/100 µl and 1 µl Annexin V-PE/100 µl (BD, #556422).

### Quantification of drug synergy

Synergy of combination treatments were assessed using MuSyC as described previously [26]. For viability end-point assays, synergistic efficacy was constrained to 0.

### Statistical analyses

R 4.0.2 [27], data.table 1.13.0 [28], ggplot2 3.3.2 [29], DEqMS 1.6.0 [30], Limma 3.44.3 [31], ggtern 3.3.0 [32], Coral [33], and superheat [34] were used for data analyses and plotting. A 4-parameter log-logistic dose–response model (drc 3.0.1) was used to fit proliferation and viability data and estimate 95% confidence [35]. *P* values were corrected for multiple comparisons using q-value estimation to control the false discovery rate (qvalue 2.20.0) [36]. Where applicable, paired *t* tests were used to account for blocking by independent replications within experiments, as described in the figure legends. Individual data points are shown when possible. Unless otherwise indicated in the figure legends, error bars indicate arithmetic mean ± standard error of the mean. The exact sample size for each experimental group/condition is stated in the figure legends. Samples sizes were predetermined heuristically. Only biological replicates were shown and used for statistical inferences. No data were excluded from analysis.

## Results

### FLT3 inhibition reshapes the nascent proteome

**Fig. 1:**
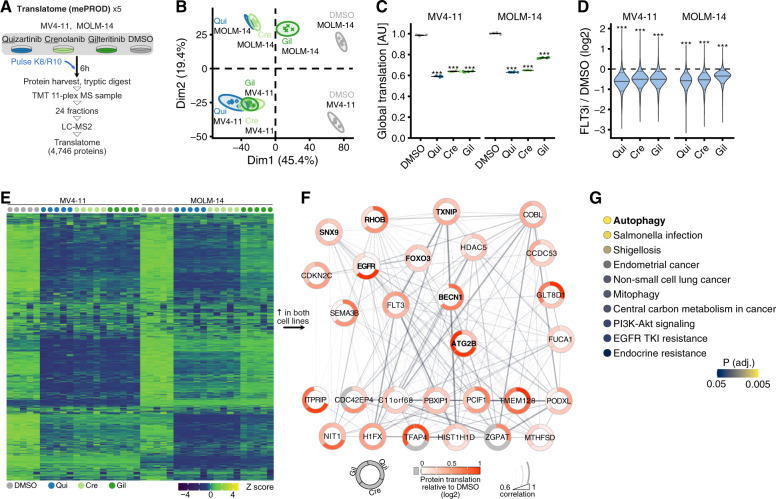
Global translatome proteomics identifies changes in the nascent proteome upon FLT3-ITD inhibition. **A** Experimental layout. FLT3-ITD + AML cell lines MV4-11 and MOLM-14 were treated with 10 nM quizartinib (Qui), crenolanib (Cre), gilteritinib (Gil) or DMSO control for 6 h (h) in Lys8- and Arg10-containing SILAC-heavy medium (*n* = 5). **B** Principal component analysis of all conditions and replicates. Dim, dimension. **C** Summed abundances of all newly translated, SILAC-heavy labeled proteins following FLT3 inhibitors (FLT3i) relative to DMSO control. Values are scaled such that the overall mean of each cell line’s DMSO condition is 1. Horizontal bars indicate mean, error bars show SEM; P values by two-sided paired *t*-test against DMSO (***P < 0.001). AU, arbitrary units. **D** Distribution of log2 fold changes (FC) relative to DMSO controls. Thick line depicts the median log2 FC, dashed lines depict the 10th/90th percentiles. *P* values were obtained by testing the log2-transformed SILAC-heavy protein abundances (averaged across replicates) between DMSO and FLT3i conditions using a two-sided paired *t*-test (***P < 0.001). **E** Heat map showing row-scaled Z scores for all measured proteins across all conditions. **F** Network of proteins upregulated in both cell lines following FLT3i. Nodes are proteins, circle segments around nodes indicate the average log2 FC for each FLT3 inhibitor, line strength between any two nodes is scaled by the pairwise Pearson correlation coefficient. Autophagy-related proteins are manually highlighted by bold typeface. **G** KEGG pathway analysis on network nodes from **F**. All significant (*P* < 0.05, adjusted for multiple testing by g:SCS) annotations are shown. See also Supplementary Fig. 1.

To identify pathways responsible for cell-autonomous resistance to FLT3i in AML, we used a recently developed unbiased proteomic method—multiplexed enhanced protein dynamics (mePROD)—to measure acute changes in the translation rate of thousands of proteins [16, 37]. mePROD proteomics quantifies translation changes by monitoring incorporation of SILAC amino acids into newly synthesized proteins, thereby enabling global, perturbation-free and unbiased analysis of the cellular response to FLT3i. FLT3-ITD + MV4-11 and MOLM-14 AML cells were treated with either vehicle (DMSO) or quizartinib, crenolanib or gilteritinib at 10 nM for 6 h (h) in SILAC-heavy (Lys8 and Arg10 labeled) medium, followed by protein isolation, tryptic digest, tandem-mass tag (TMT)-based multiplexing, offline fractionation and analysis by LC-MS^2^ (Fig. 1A). We quantified the relative abundance of newly translated, SILAC-heavy labeled proteins for 4746 proteins across all conditions and five independent replicates (Supplementary Table 1). This approach revealed global attenuation of translation and an extensive rearrangement of the cellular translatome upon FLT3 inhibition (Fig. 1B–D). Most proteins exhibited decreased translation, with distinct cell-line specific clusters (Fig. 1E and Supplementary Fig. 1A, B). By analyzing the fraction of proteins with increased translation after FLT3i treatment, we found a set of only 28 proteins that exhibited increased translation in both AML cell lines (Fig. 1F). KEGG gene set enrichment analysis revealed a cluster of autophagy-related proteins, as well as nine other pathway annotations (Fig. 1G). Autophagy-related proteins constituted the largest cluster of proteins with increased translation. We also observed a stabilization or increase in the translation rates of a large subset of the measured kinome relative to the global translation repression (Supplementary Fig. 1C), indicating that these protein kinases share a common translation program upon FLT3i. mePROD proteomics in a primary FLT3-ITD + AML sample treated ex vivo with either vehicle or quizartinib, crenolanib or gilteritinib at 10 nM for 6 h also identified increased translation of autophagy-related proteins upon FLT3i (Supplementary Fig. 2). Finally, RNA-Seq expression profiling in MV4-11 and MOLM-14 cells treated with FLT3i for 24 h revealed increased expression of autophagy-related genes, indicating that an additional transcriptional response redirects cellular state towards maintenance of autophagy under conditions of prolonged FLT3 inhibition (Supplementary Fig. 3).

### Integration with phospho-proteomics reveals autophagy as key pathway modulated by FLT3 inhibition

**Fig. 2:**
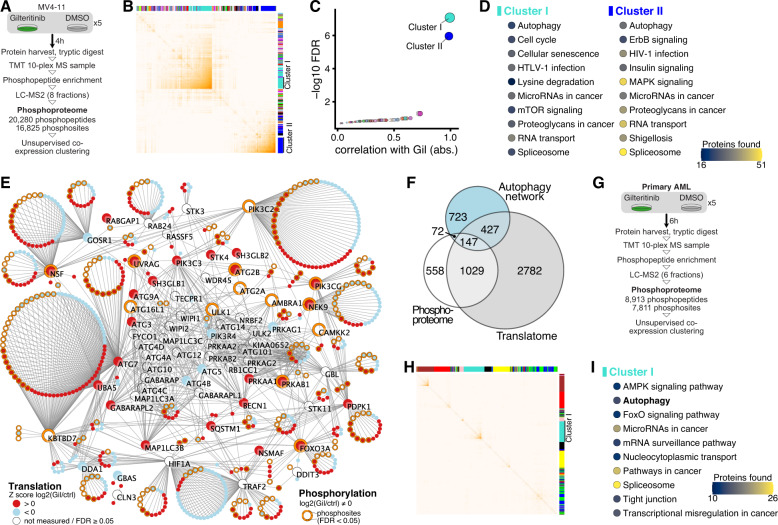
Integration with phospho-proteomics identifies the autophagy network as the major target of cellular co-regulation following FLT3-ITD inhibition. **A** Experimental scheme. MV4-11 cells were treated with 10 nM gilteritinib or DMSO control for 4 h (*n* = 5). **B** Heatmap depicting unsupervised co-expression clustering of quantified phosphosites. Individual clusters are identified by distinct colors on top row and right column. **C** Correlation (absolute) of phosphosite co-expression clusters with experimental treatment condition and associated statistical significance (FDR, false discovery rate). **D** KEGG pathway analysis on the two clusters identified by unsupervised co-expression clustering which were significantly associated with gilteritinib treatment. The top ten significant (*P* < 0.05, adjusted for multiple testing by g:SCS) pathway annotations are listed alphabetically for each cluster. **E** Translatome and phospho-proteome data (gilteritinib, MV4-11) mapped onto the network of human core autophagy proteins and their high-confidence (>0.9) STRING interactors. Core autophagy proteins are labeled. Node fill color shows significant (FDR < 0.05), global Z-scored log2 FC in protein translation. Circle segments around nodes indicate individual, significantly changing phosphosites (FDR < 0.05). **F** Euler diagram of E, showing the number of proteins with significant changes in translation and phosphorylation upon gilteritinib treatment in MV4-11 cells, mapped onto the autophagy network. **G** Experimental design. Primary FLT3-ITD + AML cells were treated with 10 nM gilteritinib or DMSO for 6 h (*n* = 5). **H** Heatmap depicting unsupervised co-expression clustering of quantified phosphosites. Individual clusters are identified by distinct colors on top row and right column. **I** KEGG pathway analysis on the main phosphosite cluster identified by unsupervised co-expression clustering. The top ten significant (*P* < 0.05, adjusted for multiple testing by g:SCS) pathway annotations are listed alphabetically.

To understand the consequences of modulated kinase levels upon FLT3i, we carried out phospho-proteomics following FLT3i treatment. MV4-11 cells were treated with either vehicle (DMSO) or gilteritinib at 10 nM for 4 h, followed by TMT-multiplexing, phosphopeptide enrichment, fractionation and analysis by LC-MS^2^ (Fig. 2A). Unsupervised co-expression clustering of the 16,825 identified and quantified phosphosites on 4125 proteins (Supplementary Table 2) revealed two main phosphosite clusters that significantly correlated with FLT3i treatment (Fig. 2B, C). Strikingly, pathway analysis on the level of individual phosphoproteins again identified autophagy among the top ten significantly enriched KEGG pathway annotations in both clusters (Fig. 2D). mTORC1 signaling, an important regulator of autophagy, was also identified (Fig. 2D). Mapping available translatome and phospho-proteome data onto the human autophagy network [38, 39], we consistently found co-regulation of translation and phosphorylation in a large subset of core autophagy proteins and their high-confidence STRING protein interactors (Fig. 2E, F). We repeated this experiment in a primary FLT3-ITD + AML sample treated either with gilteritinib or DMSO at 10 nM for 6 h (Fig. 2G) and again found autophagy among the top KEGG pathway annotations enriched in phosphoproteins clustered by unsupervised co-expression clustering (Fig. 2H, I and Supplementary Fig. 4). Together, these two complementary proteomic approaches in cell lines and primary cells identified autophagy as the key pathway modulated upon FLT3 inhibition.

### FLT3 inhibitors selectively induce autophagy in FLT3-ITD + AML cells in vitro

**Fig. 3:**
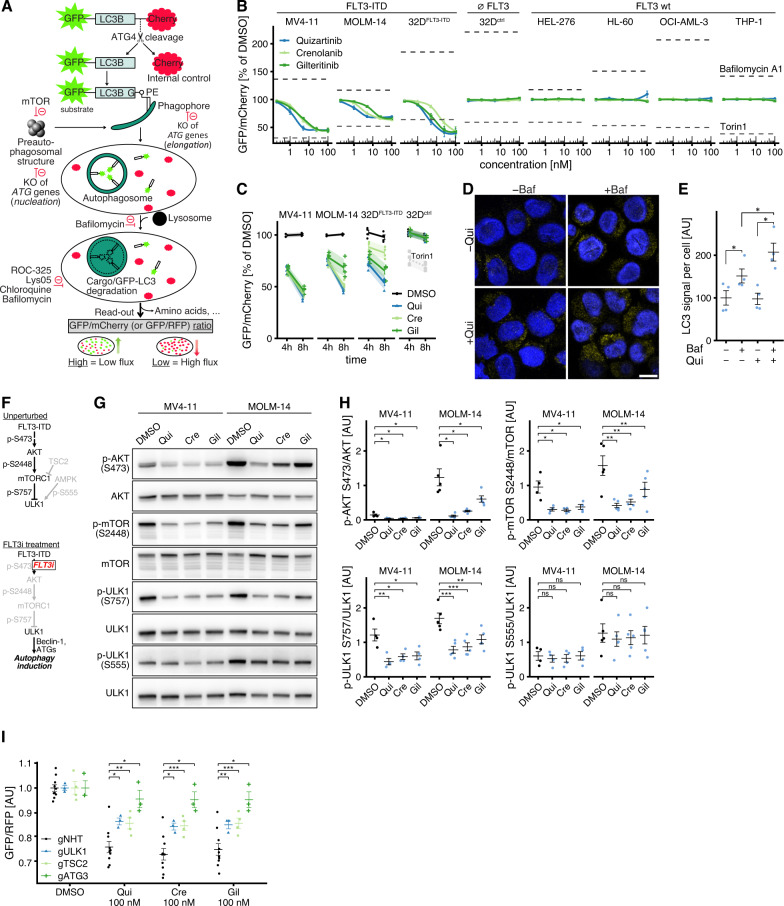
FLT3 inhibitors induce autophagy via AKT/mTORC1/ULK1 and ATG3. **A** Cells were engineered to constitutively express a ratiometric autophagic flux reporter, GFP-LC3B-mCherry or GFP-LC3B-RFP. Following cleavage by ATG4B after translation, GFP-LC3B is incorporated into nascent autophagosomes and degraded in an autophagy-dependent manner, whereas mCherry (or RFP) remains in the cytosol. A decrease in GFP/mCherry ratio indicates an increase in autophagic flux, whereas an increase in GFP/mCherry ratio is evidence of a decrease in autophagic activity. Loss of essential autophagy genes (ATGs) or lysosomal inhibitors block autophagic cargo degradation. **B** Flow cytometry measurements of GFP-LC3B/mCherry relative to DMSO control in various cells lines expressing either FLT3-ITD (MV4-11, MOLM-14, 32D-FLT3ITD; *n* = 4), FLT3 wildtype (HEL-276, HL-60, OCI-AML-3, THP-1; *n* = 3) or no FLT3 (32D-ctrl; *n* = 4) after 6 h treatment with quizartinib, crenolanib or gilteritinib at 0.2–100 nM. Bafilomycin A1 (100 nM) and Torin1 (1000 nM) served as negative and positive controls. Dots indicate mean, error bars show SEM. **C** Flow cytometry measurements of GFP-LC3B/mCherry relative to DMSO after 4 h and 8 h of treatment with 10 nM quizartinib (Qui), crenolanib (Cre) or gilteritinib (Gil) in MV4-11 (*n* = 4), MOLM-14 (*n* = 4), 32D-FLT3ITD (*n* = 4) and 32D-ctrl (*n* = 4) cells. Torin1 (100 nM) was used as a positive control. Points indicate individual measurements normalized to the mean of DMSO-treated cells, error bars denote SEM, shaded regions show 95 % confidence intervals. **D** Representative confocal microscopy images (z slices) of MV4-11 cells immunostained against endogenous LC3A/B after 100 nM quizartinib (Qui) or DMSO vehicle for 4 h, ±concurrent inhibition of autophagosomal LC3 degradation by 100 nM Bafilomycin A1 (Baf) for 4 h. The scale is identical for all images. Scale bar (lower right) measures 10 µm. **E** Quantification of cytosolic LC3 signal (*n* = 4). The accumulation of LC3 upon concurrent Bafilomycin treatment is dependent upon autophagic flux. Values are scaled such that the overall mean of untreated samples is 100. Points indicate means from individual experiments, horizontal bar denotes overall mean, error bars show SEM; *P* values by two-sided paired *t*-test (*P < 0.05). **F** Pathway diagrams showing the AKT-mTORC1-ULK1 axis with its main catalytic/regulatory phosphosites under unperturbed growth conditions (upper) and FLT3 inhibitor treatment (lower), as investigated in the next panels. **G** Representative immunoblots of lysates from MV4-11 and MOLM-14 cells treated with 10 nM quizartinib, crenolanib, gilteritinib or DMSO control for 4 h. **H** Densitometric quantification of g (MV4-11: *n* = 4; MOLM-14: *n* = 5). Horizontal bar indicates mean, error bars show SEM; *P* values by two-sided paired *t*-test (*P < 0.05, **P < 0.01, ***P < 0.001, not significant (ns) *P* ≥ 0.05). **I** Flow cytometry measurements of GFP-LC3B/RFP in MV4-11 Cas9 cells transduced with gRNA against ULK1, TSC2, ATG3 or non-human target (NHT) and treated with DMSO control or 100 nM quizartinib, crenolanib or gilteritinib for 4 h (gULK1, *n* = 3; gTSC2, *n* = 4; gATG3, *n* = 4). Values are scaled such that the overall mean of each genotype’s DMSO condition is 1. Horizontal bar indicates mean, error bars show SEM; *P* values by two-sided paired *t* test (*P < 0.05, **P < 0.01, ***P < 0.001). See also Supplementary Fig. 9.

We first confirmed that cells were viable without induction of apoptosis after short-term FLT3i treatment conditions (Supplementary Fig. 5). To functionally determine whether FLT3i induce autophagy, we constitutively expressed a ratiometric fluorescent autophagic flux reporter (GFP-LC3B-mCherry, Fig. 3A) [40] in various human and murine AML cell lines with different FLT3 status. All three FLT3i induced autophagy in a concentration-dependent manner in cells expressing FLT3-ITD (MV4-11, MOLM-14, 32D-FLT3-ITD), whereas cells expressing wildtype FLT3 (HEL-276, HL-60, OCI-AML-3, THP-1) or no FLT3 (32D-ctrl) did not show any response (Fig. 3B). Drug-induced autophagic flux further increased with treatment time (Fig. 3C). We also measured autophagic flux by quantifying the accumulation of endogenous LC3-positive punctae following autophagy inhibition by Bafilomycin A1 [41]. LC3-positive autophagosomes were significantly increased upon FLT3 inhibition with quizartinib (Fig. 3D, E and Supplementary Fig. 6), confirming our proteomics-derived hypothesis that autophagy is a key pathway induced by FLT3i.

### FLT3i-induced autophagy proceeds via AKT/mTORC1/ULK1 and requires ATG3

Our phospho-proteomics data had identified altered mTORC1 signaling (Fig. 2D) upon FLT3i. Activation of AKT/mTORC1, which is a downstream effector of FLT3-ITD signaling [3], can suppress autophagy by phosphorylating ULK1/ATG1 at S757, whereas phosphorylation of ULK1 at S555 by AMPK can induce autophagy (Fig. 3F) [42, 43]. To determine the mechanism of autophagy induction by FLT3i, we next treated MV4-11 and MOLM-14 cells with 10 nM quizartinib, crenolanib or gilteritinib for 4 h and assessed the phosphorylation status of potential mediators of autophagy induction by immunoblotting. Inhibition of FLT3-ITD signaling in MV4-11 or MOLM-14 cells led to decreased phosphorylation at AKT S473, decreased phosphorylation at the AKT substrate [44] mTOR S2448, and decreased phosphorylation at the mTORC1 substrate ULK1 S757 (Fig. 3G, H and Supplementary Fig. 7), establishing the signaling pathway leading from FLT3i to autophagy induction. We also observed decreased phosphorylation at these phosphosites (AKT S473, mTOR S2448, and ULK1 S757) in a primary FLT3-ITD + AML sample treated ex vivo with FLT3i (Supplementary Fig. 8). There was no increase in AMPK-mediated phosphorylation of ULK1 at S555 (Fig. 3G, H). However, AMPK has a diverse set of targets including other phospho-sites on ULK1 and the lack of increased ULK1 phosphorylation at S555, although one of its best-characterized targets, does not rule out additional AMPK contribution to ULK1 activation [43]. CRISPR/Cas9-targeting of TSC2, a negative mTORC1 regulator, ULK1 or ATG3, a core autophagy protein required for autophagosome formation, significantly attenuated or largely abolished autophagy induction by 10 and 100 nM quizartinib, crenolanib or gilteritinib in MV4-11 cells (Fig. 3I and Supplementary Fig. 9). Together, this shows that autophagy induction following FLT3i involves the AKT-mTORC1-ULK1 signaling axis and requires ATG3.

### Autophagy inhibition synergizes with FLT3 inhibition in vitro

**Fig. 4:**
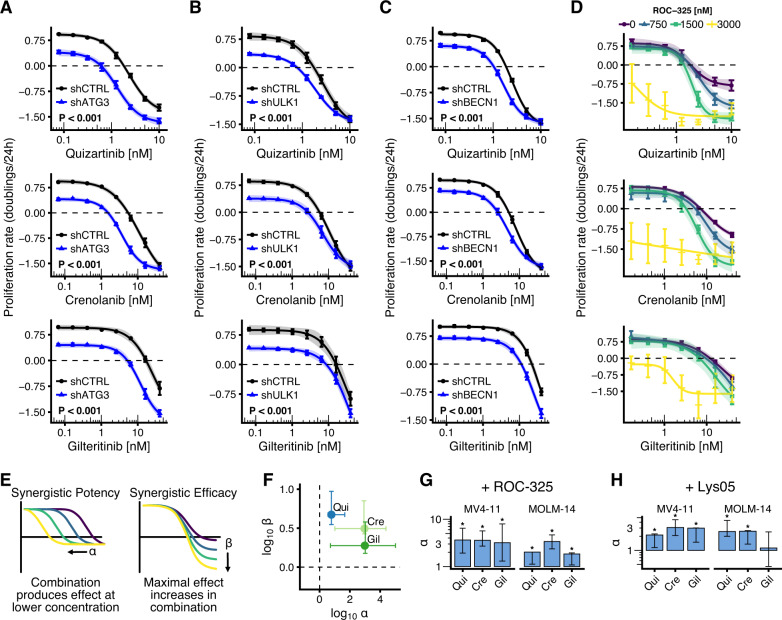
Autophagy inhibition synergizes with FLT3 inhibitors to inhibit AML cell proliferation in vitro. **A** Time-averaged proliferation rates (cell number doublings/24 h) of MOLM-14 cells transduced with shRNA against ATG3 (shATG3) or **B** ULK1 (shULK1) or **C** BECLIN1 (shBECN1) or non-human target (shCTRL) and treated with varying concentrations of quizartinib, crenolanib or gilteritinib for 72 h (*n* = 3). Cell numbers were measured every 24 h by flow cytometry. Dots indicate mean, error bars show SEM, shaded regions indicate 95% confidence interval of the four-parameter log-logistic dose-response models; *P* values by *F* test (***P < 0.001). See also Supplementary Figs. 10, 11. **D** Dose–response curves (four-parameter log-logistic function) showing time-averaged proliferation rates (cell number doublings/24 h) of MV4-11 cells co-treated with lysosomal autophagy inhibitor ROC-325 and FLT3 inhibitors (FLT3i) quizartinib, crenolanib or gilteritinib for 72 h (*n* = 3). Cell numbers were measured every 24 h by flow cytometry. Dots indicate mean, error bars show SEM, shaded regions indicate 95 % confidence interval of the dose-response models. See also Supplementary Figs. 13. **E** Scheme depicting synergistic potency (*α* > 1) and synergistic efficacy (*β* > 0) for drug combinations. **F** Quantification of synergy parameters *α* (ROC-325 potentiates FLT3i) and *β* (maximal achievable effect increases in combination) for data from C–D by MuSyC. Error bars indicate non-parametric 95 % confidence intervals. **G** Quantification of synergistic potency α from endpoint viability assays. MV4-11 and MOLM-14 cells were co-treated with ROC-325 and quizartinib, crenolanib or gilteritinib at varying concentrations and viability was measured after 72 h (*n* = 4). Error bars indicate non-parametric 95% confidence intervals. *α* values > 1 (synergistic potency) with lower confidence intervals that exclude 1 (significant synergism) are marked by an asterisk. See also Supplementary Fig. 14A. **H** Experiment as in **G**, except that cells were co-treated with lysosomal autophagy inhibitor Lys05 instead of ROC-325 (*n* = 4). See also Supplementary Fig. 14B.

Next, we hypothesized that drug-induced autophagy may be the cytoprotective mechanism explaining non-genetic primary FLT3i resistance in AML and tested the functional consequences of autophagy inhibition on the response to FLT3i. Indeed, genetic impairment of autophagy by shRNA-mediated knockdown of ATG3, ULK1 or its target Beclin1 in MV4-11 or MOLM-14 cells significantly potentiated the antiproliferative effect of quizartinib, crenolanib and gilteritinib (Fig. 4A–C and Supplementary Figs. 10, 11). We opted for shRNA knockdown instead of CRISPR/Cas9-mediated knockout to avoid potentially lethal effects and clonal selection due to loss of essential core autophagy genes [45–47]. To also evaluate pharmacological autophagy inhibitors in combination with FLT3i, we chose lysosomal autophagy inhibitors ROC-325 and Lys05, two potent and orally bioavailable chloroquine derivatives that were recently developed [48, 49]. Both compounds impaired or almost completely inhibited autophagy induction resulting from FLT3i (Supplementary Fig. 12). To assess both synergy in potency and efficacy of the combination treatments, we measured proliferation kinetics every 24 h and employed multidimensional synergy of combination analysis (MuSyC) [26] to determine synergistic potency *α* and synergistic efficacy *β*. Co-treatment of MV4-11 cells with ROC-325 and quizartinib, crenolanib or gilteritinib led to a significant and large synergy in efficacy and potency of the FLT3i (Fig. 4D–F and Supplementary Fig. 13). We then used standard 72 h endpoint viability assays that allow for more throughput, while still providing a read-out of potency. The combination of either ROC-325 or Lys05 treatment with FLT3i synergistically increased the potency of FLT3i (with the exception of Lys05+gilteritinib in MOLM-14 cells, which only displayed additive potency) (Fig. 4G, H and Supplementary Fig. 14). In contrast, cells expressing wildtype FLT3 (HEL-276, HL-60, OCI-AML-3, THP-1) co-treated with ROC-325 and quizartinib, crenolanib or gilteritinib did not exhibit any synergistic increases in potency but instead showed (non-significant) antagonism (Supplementary Fig. 15A, B). These results were corroborated in three FLT3-ITD + patient-derived xenograft (PDX) AML samples co-treated with gilteritinib and ROC-325 for 72 h. In accordance with our findings in cell lines, co-treated PDX cells showed significantly decreased viability compared to FLT3i alone (Supplementary Fig. 16A, B). Together, these results show that co-treatment with autophagy inhibitors overcomes protective FLT3i-induced autophagy and thus synergistically increases antiproliferative FLT3i effects and cell death in AML cells in vitro.

### In vivo treatment trial shows synergistic efficacy of FLT3 and autophagy inhibition in mice

**Fig. 5:**
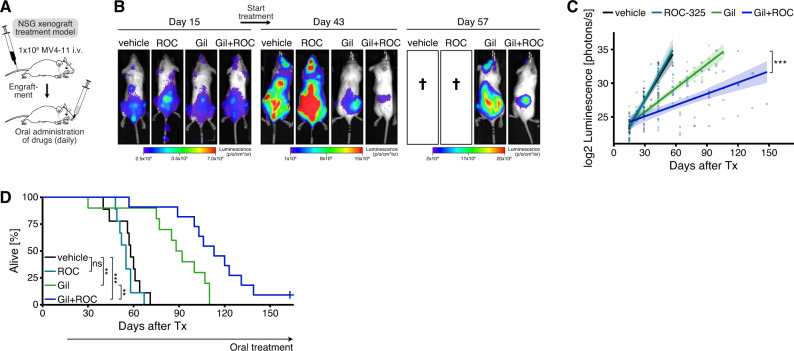
Pharmacological autophagy inhibition cooperates synergistically with FLT3 inhibition in vivo to increase antileukemic efficacy against xenografted human FLT3-ITD + AML cells in mice. **A** In vivo xenograft treatment model, schematic overview. Luciferase-labeled MV4-11 cells were transplanted into NSG (non-obese diabetic (NOD)/severe combined immunodeficient (SCID)/Il2rg−/−) mice by intravenous injection. After engraftment, mice were treated daily with [1] methylcellulose vehicle, [2] ROC-325 (ROC) 50 mg/kg, [3] gilteritinib (gil) 3 mg/kg, or [4] gilteritinib 3 mg/kg + ROC-325 50 mg/kg by oral gavage. **B** Representative serial bioluminescence images on day 15 after transplantation prior to treatment start and at later time points on-treatment. **C** Quantification of serial dorsal bioluminescence measurements during treatment. Individual measurements and group-wise log2-linear regressions are shown. Shaded regions indicate 95% confidence intervals of regression lines. *P* value by linear regression, comparing slope coefficients between gilteritinib and gilteritinib + ROC-325 group (***, *P* < 0.001). **D** Kaplan-Meier curves showing overall survival of xenografted NSG mice treated with either vehicle (*n* = 9), ROC-325 (*n* = 9), gilteritinib (*n* = 10), or gilteritinib + ROC-325 (*n* = 11). *P* values by robust Cox regression analysis (ns, not significant; **, *P* < 0.01; ***, *P* < 0.001). See also Supplementary Fig. 17.

To analyze the in vivo efficacy of FLT3 inhibition combined with autophagy inhibition, we generated an orthotopic AML xenograft model by tail vein injection of luciferase-labeled human MV4-11 cells into NSG (NOD/severe combined immunodeficient (SCID)/Il2rg−/−) mice (Fig. 5A). Following engraftment (Supplementary Fig. 17A), we treated the mice with either [1] vehicle control, [2] ROC-325 50 mg/kg, [3] gilteritinib 3 mg/kg, or [4] gilteritinib 3 mg/kg and ROC-325 50 mg/kg. Treatment was applied daily per oral gavage until death. Leukemic burden was assessed by serial bioluminescence imaging. The addition of ROC-325 to gilteritinib delayed leukemia development (Fig. 5B, C and Supplementary Fig. 17B) and significantly prolonged overall survival compared to gilteritinib alone in this aggressive leukemia model (Fig. 5D), whereas ROC-325 as a single agent was ineffective (Fig. 5B–D). Ex vivo measurements on isolated blasts showed increased autophagy in gilteritinib-treated mice, decreased autophagy in ROC-325-treated mice, and inhibition of gilteritinib-induced autophagy in mice co-treated with gilteritinib and ROC-325 (Supplementary Fig. 17C). These results confirm our prior in vitro data and strongly support our mechanistic synergism hypothesis.

### FLT3 inhibitors induce autophagy and synergize with autophagy inhibition in primary FLT3-ITD + AML patient cells

**Fig. 6:**
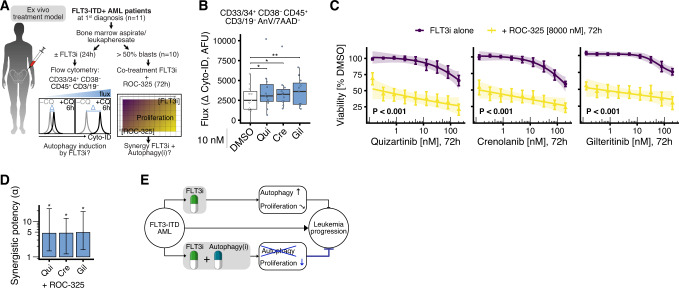
FLT3-targeted therapy induces autophagy in primary FLT3-ITD + AML patient cells and inhibition of drug-induced autophagy is synergistically cytotoxic. **A** Ex vivo treatment model, schematic overview. Primary AML cells (bone marrow aspirate or leukapheresate) obtained from untreated FLT3-ITD + AML patients at first diagnosis were assayed for autophagy induction by FLT3 inhibitors (FLT3i) and synergistic anti-proliferative effects of co-treatment with lysosomal autophagy inhibitor ROC-325 and FLT3i. Hematopoietic cytokines FLT3 ligand (FL), interleukin 3 (IL3) and stem cell factor (SCF) act as FLT3i resistance factors in vivo and were added to the culture medium. **B** Quantification of autophagic flux in primary AML cells (CD33/34+ CD38- CD45+ CD3/CD19- Annexin V/7AAD-) after 24 h treatment with 10 nM quizartinib (Qui), crenolanib (Cre), gilteritinib (Gil) or DMSO vehicle control, as measured by the difference in median Cyto-ID signal between chloroquine (CQ) co-treated cells (60 µM, last 6 h) and cells without additional CQ treatment (*n* = 11). Data are additionally summarized by boxplots (horizontal bar inside boxplot indicates median, box extends from the 25th to the 75th percentile, whiskers extend to the highest/lowest value within 1.5× inter-quartile range of the data). P values by two-sided paired *t* test (* *P* < 0.05, ** P < 0.01). **C** 72 h endpoint viability relative to DMSO vehicle control of all patient samples with AML blast percentage >50% co-treated with FLT3i and ROC-325 (*n* = 10). Dots indicate mean, error bars show SEM, shaded regions indicate 95% confidence interval of the four-parameter log-logistic dose-response models; *P* values by *F* test (*** *P* < 0.001). See also Supplementary Fig. 18. **D** Quantification of synergistic potency *α* in endpoint viability assays. Error bars indicate non-parametric 95% confidence intervals. *α* values with confidence intervals > 1 (significant synergistic potency) are marked by an asterisk. **E** Schematic representation of the combination treatment and its synergistic antileukemic action.

Finally, we aimed to validate these findings in a representative set of patient samples and obtained primary AML blasts from 11 untreated FLT3-ITD + patients at first diagnosis (Supplementary Table 3). We used a short-term ex vivo culture system with serum-free primary stem cell medium and human hematopoietic cytokines (FLT3 ligand, interleukin 3, and stem cell factor) that are present in the AML bone marrow environment and contribute to FLT3-ITD inhibitor resistance [50–52]. We tested whether FLT3i induced autophagy in these primary AML cells and whether FLT3i synergized with the lysosomal autophagy inhibitor ROC-325. To quantify autophagic flux, primary samples were treated with 10 nM FLT3i for 24 h with or without additional short-term autophagy inhibition by 60 µM chloroquine for the last 6 h [41]. We then measured the accumulation of Cyto ID-stained, undegraded autophagosomes ±chloroquine in CD45+ CD33/CD34+ CD38− CD3/CD19− AnV− 7AAD− AML blasts by flow cytometry (Fig. 6A, left). Cyto-ID, a well-established fluorescent dye, selectively labels autophagic vesicles and enables quantitative measurements in scarce samples [41, 53, 54]. We observed a significant increase in Cyto-ID staining intensity ±chloroquine for primary AML cells treated with quizartinib, crenolanib, or gilteritinib, indicating an increase of autophagic activity by FLT3i treatment (Fig. 6B).

To test the effect of combination treatment, patient samples with an AML blast percentage >50% (*n* = 10) were co-treated with lysosomal inhibitor ROC-325 and FLT3i for 72 h (Fig. 6A, right). Treatment with FLT3i alone only partially impaired AML cell viability at concentrations up to 400 nM. By contrast, the addition of ROC-325 at 8 µM increased efficacy and potency of FLT3i (Fig. 6C). Consistent with our hypothesis, we observed a synergistic 5-fold increase in the potency of FLT3i when combined with ROC-325 (Fig. 6D), confirming that autophagy inhibitors were able to overcome therapeutic resistance to FLT3i in patient samples. Direct measurement of proliferation kinetics by flow cytometry of a patient sample with near 100% blast percentage showed proliferation of vehicle-treated AML cells (Supplementary Fig. 18A, B). Treatment of this sample with FLT3i alone led to a mostly cytostatic effect, whereas the addition of ROC-325 at 2.5–5 µM increased efficacy and potency of FLT3i and achieved cytotoxic effects (Supplementary Fig. 18A, B), confirming the data obtained from the endpoint viability assays of all patients. Together, the results show that co-treatment of primary AML cells with an autophagy inhibitor potently overcomes protective FLT3i-induced autophagy and results in FLT3i dose-dependent reduction in primary AML cell viability and proliferation (Fig. 6E).

## Discussion

Global translatome proteomics is an attractive systems biology approach to directly characterize the molecular response of cancer cells to anti-proliferative drugs and identify new mechanisms by which cells cope with pharmacological perturbations. Enabled by recent technological developments [16, 37], this allows the quantification of translation dynamics in unprecedented depth and accuracy and avoids interpretative challenges associated with RNA-seq approaches [55, 56]. It is thus ideally suited to identify adaptive, primary non-genetic mechanisms of therapy resistance. Our study is the first application of this methodology in a cancer treatment model.

FLT3 inhibition caused a pronounced decrease of global translation, in agreement with inhibition of cap-dependent translation after mTORC1 inactivation [57]. Strikingly, we consistently observed increased translation of autophagy-related proteins despite the strong inhibitory effect on general translation. This specific translation response appears to serve as a cell-autonomous stress response mechanism to on-target FLT3i treatment. In addition to autophagy-related proteins, FLT3, EGFR, FOXO3 and HDAC5 were also among the proteins with increased translation. Increased total cellular FLT3 levels upon FLT3i treatment has been observed before, although the mechanism was not uncovered [6, 58, 59], and enhanced EGFR signaling has previously been identified as a resistance factor to FLT3-ITD inhibition [60]. Similarly, FOXO3-mediated transactivation of HDAC proteins was recently identified as a resistance mechanism to FLT3-ITD inhibition [14]. We also observed distinct cell-line-dependent patterns in translation rates upon FLT3i. The molecular mechanisms regulating these specific responses of the translatome upon FLT3-targeted therapy and their functional consequences beyond autophagy activation remain largely unknown. Additionally, specific changes in protein stability may affect some mePROD measurements and this constitutes another layer of cellular adaptation that remains unexplored here.

Prior work on FLT3i resistance has examined genetic mechanisms [9, 61–64] and signaling alterations [12, 60, 65–67] that mediate resistance to individual FLT3i. Point mutations within FLT3 that prevent drug binding are now well-characterized mechanisms of primary and secondary resistance to FLT3i [11, 61]. However, these genetic mechanisms fail to explain the majority of clinically observed treatment failures due to primary FLT3i treatment resistance despite on-target activity [9, 11]. Non-genetic mechanisms of intrinsic therapy resistance are increasingly being recognized as important factors contributing to treatment failure in the absence of identifiable genetic causes [11, 68]. Stress-induced autophagy is an emerging cell-autonomous therapy resistance mechanism [46, 47, 69, 70]. Our results establish protective drug-induced autophagy as a pervasive non-genetic mechanism utilized by AML cells to escape on-target FLT3i therapy. Although autophagy-mediated functions are involved in normal hematopoiesis [71] and the role of autophagy in the initial development of malignancy is complex [17], FLT3i-induced autophagy occurred specifically in FLT3-ITD-driven AML cells, thereby allowing specific intervention.

Clinical studies investigating autophagy inhibition have so far been limited by the lack of available potent autophagy inhibitors. Hydroxychloroquine, an anti-malaria drug often repurposed as an autophagy inhibitor, has shown only moderate effects as a combination agent and demonstrated insufficient potency to effectively inhibit autophagy in malignant cells at nontoxic concentrations [72, 73]. To overcome these limitations, we used ROC-325 and Lys05, two new and ten-fold more potent chloroquine derivatives which are orally bioavailable [48, 49]. Using an unbiased method to assess drug synergy [26], we demonstrated synergy in potency and efficacy of the combination of FLT3i with ROC-325 and Lys05. In mice, ROC-325 did not produce any overt toxicities aside from a reversible decrease in body weight at doses up to 50 mg/kg [48]. We likewise have not observed consistent differences in bodyweight between mice treated with ROC-325+gilteritinib vs. gilteritinib alone (Supplementary Fig. 17D). However, we have not formally assessed toxicity towards normal HSC and other organs, and more data will be required to comprehensively judge the toxicity of combined FLT3 and autophagy inhibition. Recently, ROC-325 was found to synergize with the antileukemic activity of azacytidine [74]. In our hands, ROC-325 was more potent than Lys05 in inhibiting FLT3i-induced autophagy. Thus, our results render ROC-325 a promising candidate for clinical translation and provide a rationale for safety and efficacy evaluation of ROC-325 in combination with 2nd generation FLT3 inhibitors such as gilteritinib.

Our current analyses of treatment efficacy in PDX and primary AML cells ex vivo was limited to the bulk blast cell population and, due to limited sample amounts, we do not have NGS data on clonal architectures available for primary samples. Given the limited predictive power of cellular in vitro and ex vivo models as well as cell-line derived mouse model, future preclinical work will need to demonstrate efficacy in genetically heterogeneous models of FLT3-ITD + AML with realistic (sub-)clonal architectures such as FLT3-ITD + PDX cells in NSG mice. This will also allow a more precise delineation of treatment efficacy in primary AML cell subpopulations such as progenitor and stem cells. Further work will also be required to elucidate the role of autophagy in cases of *acquired* FLT3i resistance without identifiable resistance-mediating genetic alterations.

Taken together, our systems biology approach to study the global translatome and phospho-proteome showed that the autophagy network escapes translational repression and functions as a cell-autonomous, non-genetic primary resistance pathway against FLT3i treatment. We validated these findings in cellular models, in an in vivo treatment model and in a representative set of primary AML patient samples. Our preclinical data thus suggest a treatment strategy that combines targeted FLT3 inhibition with inhibition of drug-induced autophagy for the treatment of FLT3-ITD + AML. This provides a rational roadmap to increase the clinical potency and efficacy of FLT3i, opens new perspectives for the study of translation dynamics in cancers and may serve as a blueprint for future functional translatome proteomics studies to systematically investigate cell-autonomous therapy resistance mechanisms.

## Supplementary information

Supplementary Data

Supplementary Table 1

Supplementary Table 2

## Data Availability

The datasets supporting the conclusions of this article are available from the ProteomeXchange Consortium via the PRIDE [75] repository with the dataset identifiers PXD023125, PXD023123, PXD034080, and PXD034081. For detailed information regarding mass spectrometry proteomics, proliferation assays, lentiviral transduction, constructs, and immunoblotting, see Supplementary Materials and Methods.
