# Correction: Translatome proteomics identifies autophagy as a resistance mechanism to on-target FLT3 inhibitors in acute myeloid leukemia

**DOI:** 10.1038/s41375-022-01721-y

**Published:** 2022-11-08

**Authors:** Sebastian E. Koschade, Kevin Klann, Shabnam Shaid, Binje Vick, Jan A. Stratmann, Marlyn Thölken, Laura M. Meyer, The Duy Nguyen, Julia Campe, Laura M. Moser, Susanna Hock, Fatima Baker, Christian T. Meyer, Frank Wempe, Hubert Serve, Evelyn Ullrich, Irmela Jeremias, Christian Münch, Christian H. Brandts

**Affiliations:** 1 Department of Medicine, Hematology/Oncology, University Hospital, Goethe University Frankfurt, Frankfurt, Germany; 2 Institute of Biochemistry II, Faculty of Medicine, Goethe University Frankfurt, Frankfurt, Germany; 3 German Cancer Consortium (DKTK), Heidelberg, Germany; 4 German Cancer Research Center (DKFZ), Heidelberg, Germany; 5 Frankfurt Cancer Institute, Goethe University Frankfurt, Frankfurt, Germany; 6 University Cancer Center Frankfurt (UCT), University Hospital, Goethe University Frankfurt, Frankfurt, Germany; 7 Helmholtz Zentrum München, German Research Center for Environmental Health, Research Unit Apoptosis in Hematopoietic Stem Cells, Munich, Germany; 8 Department for Children and Adolescents, Experimental Immunology, University Hospital, Goethe University Frankfurt, Frankfurt, Germany; 9 Department for Children and Adolescents, Division for Stem Cell Transplantation, Immunology and Intensive Care Medicine, University Hospital, Goethe University Frankfurt, Frankfurt, Germany; 10 Vanderbilt University, Center for Cancer Systems Biology at Vanderbilt, Nashville, TN USA; 11 Department of Pediatrics, Dr. von Hauner Children’s Hospital, University Hospital, LMU Munich, Munich, Germany; 12 Cardio-Pulmonary Institute, Frankfurt, Germany

**Keywords:** Acute myeloid leukaemia, Cancer therapeutic resistance, Preclinical research

Correction to: *Leukemia* 10.1038/s41375-022-01678-y, published online 23 August 2022

Due to a technical problem, Figures 2, 3 and 6 were not displayed correctly in the original article. The original article has been corrected.

