# Supplementary material for: Translatome proteomics identifies autophagy as a resistance mechanism to on-target FLT3 inhibitors in acute myeloid leukemia
Source: Leukemia. 2022 Aug 23;36(10):2396–407. doi: 10.1038/s41375-022-01678-y (PMC9522593; doi:10.1038/s41375-022-01678-y)
Supplement: Supplementary file 1 — Supplementary Data [file 41375_2022_1678_MOESM1_ESM.pdf]

SUPPLEMENTARY DATA

Translatome proteomics identifies autophagy as a resistance mechanism to on-target FLT3 inhibitors in acute myeloid leukemia

Sebastian E. Koschade, Kevin Klann, Shabnam Shaid, Binje Vick, Jan A. Stratmann, Marlyn Thölken, Laura M. Meyer, The Duy Nguyen, Julia Campe, Laura M. Moser, Susanna Hock, Fatima Baker, Christian T. Meyer, Frank Wempe, Hubert Serve, Evelyn Ullrich, Irmela Jeremias, Christian Münch* & Christian H. Brandts*

List of supplementary data: Supplementary Materials and Methods, Supplementary Figures 1–18, Supplementary Source Figure Immunoblots (PDF), Supplementary Tables 1 & 2 (Excel), 3

Supplementary Materials and Methods

Study design

Previous studies have firmly established the relevance of constitutively activated FLT3 as a therapeutic target. However, about half of FLT3-ITD+ AML patients fail to respond to 2nd generation FLT3 inhibitors despite lacking known genetic mutations conferring primary resistance to FLT3 inhibitors. The objective of this study was to identify a cell-autonomous non-genetic mechanism of primary resistance by which FLT3-ITD+ AML cells can oppose on-target FLT3 kinase inhibitor treatment. Functional mass spectrometry techniques were used to globally profile cellular responses to the 2nd generation FLT3 inhibitors quizartinib, crenolanib and gilteritinib. The expectation was that newly developed translation proteomics would enable direct measurements of cellular drug responses and identify therapy resistance mechanisms. These assays pinpointed autophagy as a major stress response pathway activated upon FLT3 inhibitor treatment. *In vitro*, *in vivo* and *ex vivo* follow-up studies utilizing flow cytometry, fluorescence microscopy, immunoblotting, shRNA, and CRISPR/Cas9 techniques were performed on human and murine AML cell lines, human AML cells xenografted into NSG mice, human AML patient-derived xenografts and human primary cells collected from untreated newly diagnosed AML patients. The exact sample size for each experimental group/condition are stated in the figure legends. Samples sizes were predetermined heuristically. Investigators were not blinded for the experiments. Only biological replicates were shown and used for statistical inferences. No data was excluded from analysis. All animals assigned to treatment groups were included in the analysis. Animals were randomized to treatment groups. Randomization was not performed for cellular assays. Reproducibility

of results has been assessed by independent biological replicates. Random technical variation was generally controlled by averaging technical replicates if available, and by parallel technical sample preparation and TMT-based sample multiplexing. LC-MS batch sampling effects were corrected by internal reference scaling.

Mass spectrometry sample preparation

After lysis, lysates were precipitated with three volumes of ice-cold methanol, one volume chloroform and 2.5 volumes ddH2O. After centrifugation at 14,000 g for 45 min at 4 °C, the upper aqueous phase was aspirated and three volumes of ice-cold methanol were added. Samples were mixed and proteins pelleted by centrifugation at 14,000 g for 5 min at 4 °C. Supernatant was discarded and pellets were washed once with ice-cold methanol. Protein pellets were dried at room temperature and then resuspended in 8 M Urea, 10 mM EPPS pH 8.2 and 1 mM $CaCl_2$. Protein concentrations were determined using µBCA assay (Thermo Fisher Scientific). 140 µg of protein (translatome) or 400 µg of protein (phospho-proteome) per sample were then diluted to 1 M urea in 10 mM EPPS pH 8.2 and 1 mM CaCl2 and incubated overnight with LysC (Wako Chemicals, Osaka, Japan) at 1:50 (w/w) ratio and Trypsin (Promega) at 1:100 (w/w) ratio. Digests were acifidied using trifluoroaceticacid (TFA) to a pH of 2–3 and peptides purified using tC18 SepPak columns (50 mg, Waters, Milford, MA, USA) according to the manufacturer’s protocol. Eluates were dried by vacuum centrifugation and stored for further processing. Peptides were resuspended in TMT-labeling buffer (0.2 M EPPS pH 8.2, 10% acetonitrile) and peptide concentration determined by µBCA. 20 µg of peptides (translatome) or 125 µg of peptides (phospho-proteome) were mixed with TMT reagents (Thermo Fisher Scientific) in 1:2 (w/w) ratio and incubated for one hour at room temperature. For translatome analysis, a SILAC-heavy labeled (booster channel) and SILAC-light labeled (noise channel) HeLa digest was included in each multiplex. Subsequently, reactions were quenched by addition of hydroxylamine to a final concentration of 0.5 % at room temperature for 15 min. Samples were pooled in equimolar ratios for each channel as determined after a single injection measurement by LC-MS/MS, acidified with TFA to a pH of 2–3, and purified using Empore C18 (Octadecyl) resin material (3M Empore). C18 material was activated by incubation with methanol for 5 min, followed by one wash each with 70 % acetonitrile/0.1 % TFA and 5 % acetonitrile/0.1 % TFA. The pooled and acidified samples were loaded onto resin material and peptides were subsequently washed with 5 % acetonitrile/0.1 % TFA and eluted with 70 % acetonitrile. For translatome proteomics, the peptides were then dried for offline HPLC fractionation. For phospho-

proteomics, 1 mg of pooled peptides were used for phosphopeptide enrichment by High-Select Fe-NTA Phosphopeptide enrichment kit (Thermo Fisher Scientific) following the manufacturer's instructions. After enrichment, peptides were dried and resuspended in 70 % acetonitrile/0.1 % TFA and filtered through a C8 (Empore, Thermo Fisher Scientific) stage tip to remove contaminating Fe-NTA particles. Dried phosphopeptides were then fractionated on a C18 (Empore, Thermo Fisher Scientific) stage tip. For fractionation, C18 stage tips were washed with 100 % acetonitrile twice, followed by equilibration with 0.1 % TFA. Peptides were loaded in 0.1 % TFA and washed with water. Elution was performed with step-wise increasing acetonitrile concentrations in 0.1 % triethylamine solution (5 %, 7.5 %, 10 %, 12.5 %, 15 %, 17.5 %, 20 %, 50 %). The eight fractions were then dried for LC-MS by vacuum centrifugation. Phosphopeptides of primary cells were likewise eluted with increasing acetonitrile concentrations in 0.1 % triethylamine solution (5 %, 7.5 %, 10 %, 12.5 %, 15 %, 17.5 %, 20 %, 22.5 %, 25 %, 27.5 %, 30 %, 50 %), the twelve fractions were then concatenated into 6 fractions and dried for LC-MS.

Offline high-pH reverse phase fractionation

Peptides were fractionated using a Dionex Ultimate 3000 analytical HPLC. 240 µg of pooled and purified TMT-labeled samples were resuspended in 10 mM ammonium bicarbonate (ABC), 5 % acetonitrile (ACN) and separated on a 250 mm long C18 column (Waters XBridge C18, 4.6 mm x 250 mm, 3.5 µm particle size) using a multistep gradient from 100 % solvent A (5 % AC, 10 mM ABC in water) to 60 % solvent B (90 % CAN, 10 mM ABC in water) over 70 min. Eluting peptides were collected every 45 s for a total of 96 fractions, which were then cross-concatenated into 24 fractions and dried for further processing.

Liquid chromatography mass spectrometry

For translatome analysis, mass spectrometry data were acquired in centroid mode on a QExactive HF mass spectrometer hyphenated to an Easy nLC 1200 nano HPLC system using a nanoFlex ion source (Thermo Fisher Scientific) applying a spray voltage of 2.3 kV. Peptides were separated on a self-made, 35 cm long, 75 µm ID fused-silica column, packed in-house with 1.9 µM C18 particles (ReproSil-Pur, Dr. Maisch, Ammerbuch, Germany) and heated to 50 °C using an integrated column oven (Sonation, Biberach, Germany). HPLC solvents consisted of 0.1 % formic acid in water (buffer A) and 0.1 % formic acid, 80 % acetonitrile in water (buffer B). Each peptide fraction was eluted by a non-linear gradient from

5 %–30 % B over 90 min, followed by a stepwise increase to 95 % B in 6 min, which was held for another 9 min. Full scan MS spectra (350–1400 m/z) were acquired at a resolution of 120,000 at m/z 200, a maximum injection time of 100 ms and an automatic gain control (AGC) target value of $3x10^6$. The 20 most intense precursors per full scan with a charge state between 2 and 6 were isolated using a 1 Th window and fragmented using higher energy collisional dissociation (HCD, normalized collision energy (NCE) of 35%). MS/MS spectra were acquired with a resolution of 45,000 at m/z 200, a maximum injection time of 86 ms and an AGC target value of $1x10^5$. Dynamic exclusion was set to 20 s to limit repeated sequencing of previously acquired precursors.

For phosphopeptide analysis, mass spectrometry data were acquired in centroid mode on an Orbitrap Fusion Lumos mass spectrometer hyphenated to an Easy nLC 1200 nano HPLC system using a nanoFlex ion source (Thermo Fisher Scientific) applying a spray voltage of 2.6 kV with the transfer tube heated to 300 °C and a funnel RF of 30%. Internal mass calibration was enabled (lock mass 445.12003 m/z). Peptides were separated on a self-made, 35 cm long, 75 µm ID fused-silica column, packed in-house with 1.9 µM C18 particles (ReproSil-Pur, Dr. Maisch) and heated to 50 °C using an integrated column oven (Sonation). HPLC solvents consisted of 0.1 % formic acid in water (buffer A) and 0.1 % formic acid, 80 % acetonitrile in water (buffer B). Each peptide fraction was eluted by a non-linear gradient from 3 %–35 % B over 120 min, followed by a stepwise increase to 90 % B in 6 min, which was held for another 9 min. Full scan MS spectra (350–1400 m/z) were acquired at a resolution of 120,000 at m/z 200, a maximum injection time of 100 ms and an AGC target value of $4x10^5$. The 20 most intense precursors per full scan with a charge state between 2 and 5 were selected for fragmentation, isolated with a quadrupole isolation window of 0.7 Th and fragmented via HCD, applying a NCE of 38 %. MS/MS were acquired in the Orbitrap with a resolution of 50,000 at m/z 200, a maximum injection time of 86 ms and an AGC target value of $1x10^5$. Dynamic exclusion was set to 60 s and 7 ppm to limit repeated sequencing of already acquired precursors and advanced peak determination was deactivated.

Mass spectrometry data analysis

Raw files were analyzed using Proteome Discoverer (PD) 2.4 (Thermo Fisher Scientific). Spectra were selected using default settings. For total proteome/translatome analysis, database searches were performed using SequestHT node in PD against trypsin-digested *Homo sapiens* SwissProt database (TaxID:9606, version 2018-11-21) with static modifications set as TMT6 (N-terminal,

+229.1629)/TMTpro (N-terminal, +304.2071), and carbamidomethyl (Cys, +57.021464). Dynamic modifications were set as methionine oxidation (Met, +15.995), acetylation (protein N-terminus, +42.0.11), methionine loss (protein N-terminus, -131.040), methionine loss with acetylation (protein N-terminus, -89.030), TMT6 (Lys, +229.1629)/TMTpro (Lys, 304.2071), TMT+K8 (Lys, +237.177)/TMTpro+K8 (Lys, +312.221) and Arg10 (Arg, +10.008). For phosphopeptides, database searches were performed using SequestHT node in PD against trypsin-digested *Homo sapiens* SwissProt database (TaxID:9606, version 2020-03-12) with static modifications set as TMT6 (N-terminal, +229.1629), TMT6 (Lys, +229.1629) and carbamidomethyl (Cys, +57.021464). Dynamic modifications were set as methionine oxidation (Met, +15.995), acetylation (protein N-terminus, +42.0.11) and phosphorylation (Ser/Thr/Tyr, +79.966). After search, identifications were validated using a concatenated target-decoy strategy and FDR was estimated using q-values calculated by Percolator, applying 1 % and 5 % cut-offs for high and medium confidence hits. Only high-confidence hits were accepted for further analysis. Phosphosite localization probabilities were calculated using the ptmRS node in PhosphoRS mode and default settings. Consensus workflow for reporter ion quantification was performed with default settings, except the minimal signal-to-noise ratio was set to 5 for translatome analysis. Results were exported as Excel files for further processing.

Excel files were used as input for a custom-made in-house R pipeline. R version 4.0.2 was used together with data.table 1.13.0. Excel files with PSM data were read in and sample loading normalization was performed by total intensity normalization to the lowest channel. For mePROD analysis of translation, all possible modification states/PSMs containing a heavy label were extracted. Baseline subtraction was performed on PSM level by subtracting the measured intensities of the non-SILAC-labeled sample from all other channel values (1). Negative intensities were treated as zero. PSM data were then collapsed onto protein-level by intensity summation of PSMs belonging to the same unique protein. Internal reference scaling (IRS) (2) to one physically identical bridge channel included in all TMT multiplexes was applied to correct for LC/MS sampling batch effects. For this, intensity values were geometric-averaged across the bridge channels from individual multiplexes for each protein, the IRS normalization factor for each multiplex and protein was calculated by dividing this target value by the bridge channel intensity value, and individual protein intensities were scaled by multiplication with each protein's IRS factor per multiplex.

RNA-Seq data analysis

Illumina paired-end sequencing run files obtained from NCBI Sequence Read Archive were pseudo-aligned to the human reference transcriptome and reads were quantified using kallisto 0.46.1 (3) (100 bootstrap samples, default seed, sequence-based bias correction enabled). The transcriptome index for kallisto (k-mer default 31) was built from the human reference transcriptome GRCh38 (hg38) release 105 obtained from Ensembl. Differential expression analyses of transcripts and aggregation to the gene level was done with sleuth 0.30.0 (4), using the counts-aggregation method to obtain gene-level mean fold changes for pairwise contrasts and adjusted q-values from P values (likelihood ratio test) aggregated using lancaster method for gene-level pairwise contrasts, both as implemented in sleuth.

Generation of transgene cells

Lentivirus was generated by co-transfecting Lenti-X 293T cells with indicated vectors, psPAX2 and pMD2G plasmids using polyethylenimine. Medium was exchanged after 16 h and viral supernatant was harvested 72 hours after transfection, aliquoted and stored at −80 °C. Cells were transduced by spinfection (300 g for 1 h at 34 °C, 6.25 µg/ml polybrene). The next day, medium was exchanged and 72h after transduction, cells were selected by the addition of appropriate antibiotics (2 µg/ml puromycin; 8 µg/ml blasticidin; 300 µg/ml hygromycin). Cells were not selected for single clones and experiments were performed on bulk cell populations. 32Dcl3 cells were retrovirally transduced with human FLT3-ITD (12 aa insertion between wildtype FLT3 aa595 and aa596) in pAULO vector (5). To generate autophagic flux reporter cells, cells were lentivirally transduced with eGFP-hLC3B$^{1—120}$-mCherry-P2A-puromycin or GFP-LC3B-RFP-P2A-hygromycin at low multiplicity (MOI < 0.1). Transduction efficiency was measured by flow cytometry 72 h after transduction For targeted deletions, cells were further lentivirally transduced with a lentiCRISPRv2-based expression vector at MOI < 0.1 to express SpCas9-P2A-blasticidin, and then with lentiviral gRNA vectors. shRNA was similarly introduced by lentiviral transduction of parental cells and puromycin selection.

Constructs, gRNA and shRNA

pBABE-puro mCherry-EGFP-LC3B was a gift from Jayanta Debnath (Addgene plasmid # 22418). pMRX-IP-GFP-LC3-RFP was a gift from Noboru Mizushima (Addgene plasmid # 845739). lentiCRISPRv2 was a gift from Feng Zhang (Addgene plasmid #52961). For stable expression of autophagy flux reporter, lentiCRISPRv2 was modified by removal of the U6 promoter, gRNA spacer and

the SpCas9 coding sequence was replaced with EGFP-LC3B-mCherry using standard cloning techniques. For stable expression of autophagy flux reporter with Cas9, lentiCRISPRv2 was modified by removal of the U6 promoter, gRNA spacer, exchange of the SpCas9 coding sequence with GFP-LC3-RFP and replacement of the puromycin cassette with a hygromycin cassette. For stable SpCas9 expression, lentiCRISPRv2 was modified by removal of the U6 promoter, gRNA spacer and the puromycin resistance gene was replaced by blasticidin resistance gene.

For gRNA expression, single guide RNAs for ATG3 (#1: 5'-aggtgtaattaccccagaag-3', #2: 5'-gtagatacatatcacaacac-3'), ULK1 (5'- accccgagcttgggtacga -3'), TSC2 (5'-agcacgcagtggaagcactc-3') and NHT (#1: 5'-aaaaagcttccgcctgatgg-3'; #2: 5'-aaaacaggacgatgtgcggc-3'; #3: 5'-aaaacatcgaccgaaagcgt-3') were cloned into pKLV2.2-h7SKgRNA-hU6gRNA-PGKpuroBFP (a gift from Kosuke Yusa; Addgene plasmid #72666) at the U6 promoter site by golden gate assembly using BbsI.

The following shRNAs were used: ULK1 (Sigma-Aldrich; TRCN0000342643, Sequence 5'-CCGGACATCGAGAACGTCACCAAGTCTCGAGACTTGGTGACGTTCTCGATGTTTTTTG-3'), ATG3 (Sigma-Aldrich; TRCN0000148120, Sequence 5'-CCGGGATGTGACCATTGACCATATTCTCGAGAATATGGTCAATGGTCACATCTTTTTTG-3'), BECLIN1 (Sigma-Aldrich; TRCN0000310521, Sequence 5'-CCGGCCGACTTGTTCCTTACGGAAACTCGAGTTTCCGTAAGGAACAAGTCGGTTTTTG-3'). Non-targeting shRNA (Sigma-Aldrich; SHC002; Sequence 5'-CCGGCAACAA GATGAAGAGCACCAACTC-3') was used as a control.

For lentiviral packaging pMD2.G (Addgene #12259) and psPAX2 (Addgene #12260) were used

Autophagy assay by confocal microscopy imaging: Image acquisition and analysis

Images were acquired at a resolution of 512 x 512 pixels and a z slice distance of 1 µm. Cytosolic LC3 signal intensity was integrated per cell. Quantification analysis of LC3 stains was performed using NIH ImageJ 2.0.0 (6) and Bio-Formats plugin (7). At least 30-100 cells per experimental condition and replicate were analyzed and counted. All individual z sections of images with multiple cells were analyzed to obtain the total cytosolic LC3 signal intensity integrated per cell after image background subtraction and subtraction of nuclear, DAPI-overlapping signal.

Viability and proliferation assays

Cells were seeded into white 96-well plates (10,000 cells/well) and treated with serial dilutions of active compounds or vehicle. After 72 hours, cells were lysed with CellTiter-Glo reagent (Promega) and luminescence was measured with a Infinite 200 Pro plate reader and i-control 1.11 (Tecan). To measure proliferation, live cells were counted at the time of seeding into U-bottom 96-well plates and every 24 hours for 72 hours by removing an aliquot from the culture plate into a measurement plate, followed by addition of 7-AAD (BD) per the manufacturer's instructions and read-out on a FACSCelesta (BD) equipped with a high-throughput sampler, which allowed the analysis of a defined volume of cell suspension. Each well was mixed 5 times (50 µl volume, 200 µl/s speed) and 50 µl were acquired per well at 3 µl/s. Negative control wells without cells were included on each measurement plate. Cells were gated by forward and sideward scatter and viable (7-AAD-negative cells) were used to calculate viable cell density, total number of viable cells, and the drug-induced proliferation rate (8). Cells treated with ROC-325 were gated by forward and sideward scatter due to spectral overlap of 7-AAD with ROC-325. Proliferation doublings were calculated per well for all available time points (every 24 h). The drug-induced proliferation rate over the available time points was then calculated for each condition after averaging technical replicates per plate by fitting a linear regression model to the cell population doublings versus time (8).

Patient-derived xenograft (PDX) AML cells

AML PDX cells lentivirally transduced with firefly luciferase were transplanted into NSG (non-obese diabetic (NOD)/severe combined immunodeficient (SCID)/Il2rg−/−) mice (The Jackson Laboratory, Bar Harbour, ME, USA), isolated from bone marrow or spleen at advanced leukemia as described previously (9) and cryopreserved. After thawing, cells were cultured for 42 h prior to treatments as described for primary AML cells, except for additional supplementation of 1% penicillin/streptomycin and 10 ng/ml human thrombopoetin (STEMCELL Technologies). Firefly luciferase expression was measured using ONE-Glo EX (Promega) following the manufacturer's recommendations.

Flow cytometry

All samples were analyzed on a LSRFortessa cytometer (BD) or a FACSCelesta (BD) equipped with a HTS unit. Measurements and analyses were performed using FACSDiva software 7.0 (BD) and FlowJo software 10.7.1 (Tree Star). Instrument setup including fluorescence amplification and compensation

was performed by applying FACSDiva compensation setup. Flow cytometer performance was checked regularly using CS&T beads (BD).

Immunoblotting

Cells were lysed in modified RIPA buffer (50 mM Tris-HCl, pH 7.4; 150 mM NaCl; 1 mM EDTA; 1 % NP-40, 0.25 % Na-deoxycholate; 2x Roche EDTA-free cOmplete Protease Inhibitor Cocktail; 1 mM Na_3VO_4; 1 mM PMSF) for 30 min on ice. Lysates were cleared (13,000 g, 10 minutes), protein concentrations were measured by BCA assay (Thermo Fisher Scientific), 20 µg of protein in NuPAGE LDS sample buffer (Thermo Fisher Scientifc) with 50 mM DTT were loaded per lane of 4-12 % Bis-Tris polyacrylamide gels, separated by SDS-PAGE (70 V for 15 min, 130 V for ~2 h, constant voltage; MOPS SDS running buffer) and wet-transferred (NuPAGE transfer buffer, 20 % MeOH; 30 V, 2 h) onto 0.45 µM nitrocellulose membranes (Bio-Rad). Total protein reversibly stained with 0.00005 % (w/v) Fast Green FLC (Sigma-Aldrich, #F7252) in 6.7% (v/v) CH_3COOH/30% (v/v) MeOH (10) was detected at 700 nM with Odyssey Fc and Image Studio Software 3.1.4 (LI-COR). Membranes were blocked (5 % milk powder and 0.45 % Tween-20 in TBS; 1 h, room temperature (RT)), probed with primary antibody (4 °C, over night), followed by secondary antibody (1 h, RT). Protein bands were visualized by chemiluminescence (SuperSignal West Femto, Thermo Fisher Scientific) and detected by Odyssey Fc. For phosphoproteins, membranes were stripped with Restore PLUS (Thermo Fisher Scientific; 15 min, RT), reblocked, and reprobed for total protein species. Total protein (lanes) and specific protein bands were quantified by densitometry using Image Studio Lite 5.2.5 (LI-COR). Primary antibodies used were mouse anti-β-Actin mAb (Sigma, #A5441, AC-15; 1:10,000), rabbit anti-pULK1 S757 mAb (Cell Signaling Technology, #6888; D7O6U; 1:1,000), rabbit anti-pULK1 S555 mAb (Cell Signaling Technology, #5869, D1H4; 1:1,000), rabbit anti-ULK1 mAb (Cell Signaling Technology; #8054, D8H5; 1:1,000), rabbit anti-p-mTOR S2448 mAb (Cell Signaling Technology, #5536, D9C2; 1:1,000,), rabbit anti-mTOR mAb (Cell Signaling Technology, #2983, 7C10; 1:1,000), rabbit anti-pAKT S473 mAb (Cell Signaling Technology, #4060, D9E; 1:1,000), rabbit anti-AKT pAb (Cell Signaling Technology, #9272; 1:1,000,), rabbit anti-TSC2 mAb (Cell Signaling Technology, #4308, D93F12; 1:1,000), mouse anti-ATG3 mAb (Santa Cruz, #sc-393660, A-3; 1:500). Secondary antibody conjugates used were HRP goat anti-mouse $F(ab')_2$ IgG pAb (Jackson ImmunoResearch Laboratories, #115-036-062, RRID AB_2307346; 1:10,000) and HRP goat anti-rabbit $F(ab')_2$ IgG pAb (Jackson ImmunoResearch Laboratories, #111-036-144, RRID AB_2337946; 1:10,000).

Network and enrichment analysis

Cytoscape 3.8.1 (11) was used for network visualization. For KEGG pathway analyses, gene sets were extracted from data using fold change and significance cut-offs and submitted to g:GOsT (multiple testing correction by g:SCS, known domain scope) (12). Co-expression clustering was done by weighted gene correlation network analysis (13) implemented in R's WGCNA 1.69. Phosphopeptide abundances were summed per unique site, and sites with quantifications in all channels entered analysis. The adjacency matrix was constructed by calculating all pairwise, signed Pearson correlation coefficients, soft-thresholded with a power of 10 (MV11)/16 (primary AML) (for which the scale-free topology index reached 0.9) to yield an undirected adjacency matrix and transformed into a signed topological overlap matrix. Clusters were identified by hierarchical clustering with dynamic tree cut and clusters with a eigengene correlation > 0.8 were merged. Merged clusters were related to the experimental condition by calculating the Pearson correlation between module eigengenes and an indicator variable encoding treatment.

Supplementary Figures

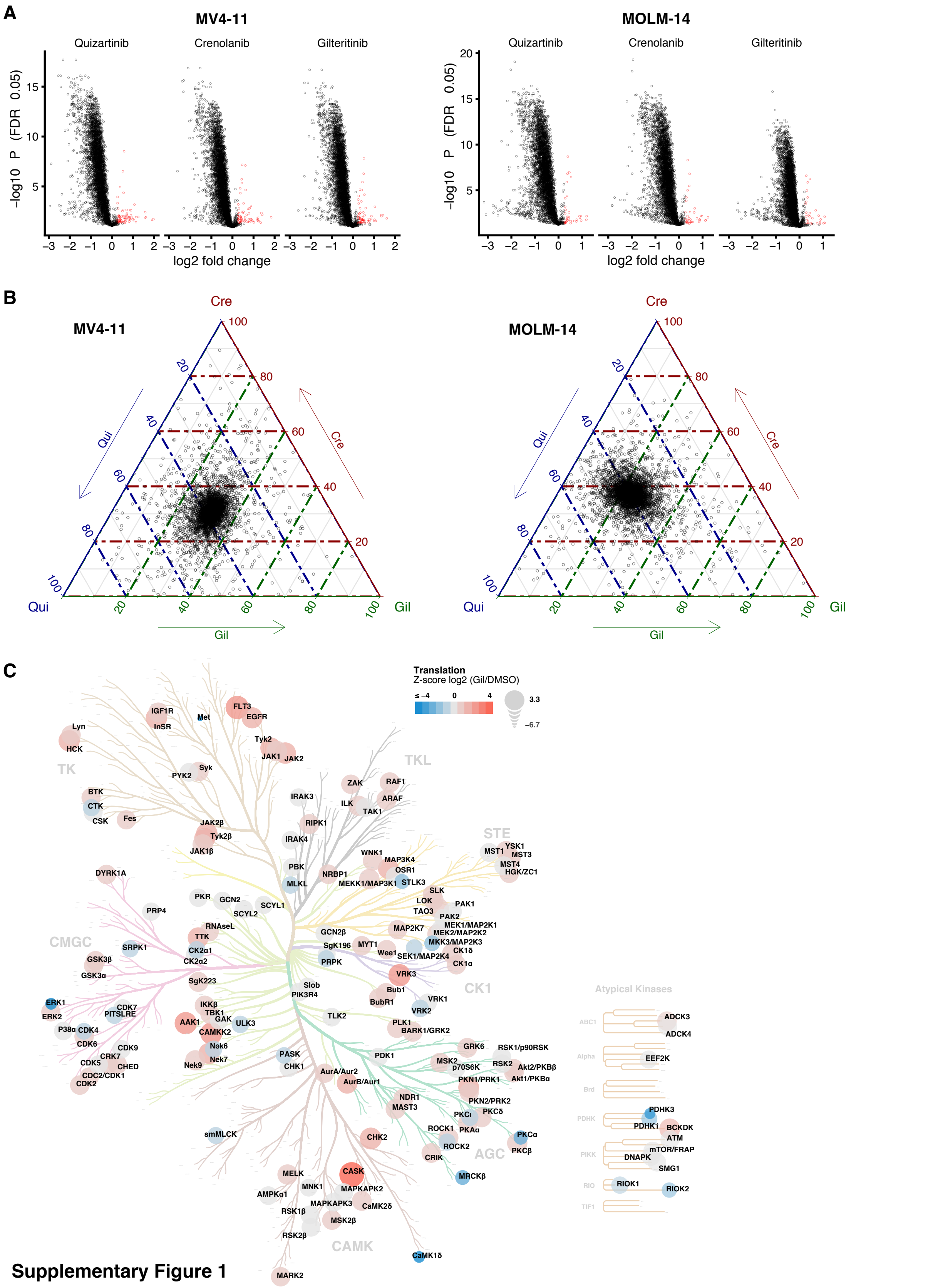

Supplementary Figure 1

Supplementary Fig. 1: Differential expression analysis of protein translation in cell lines following FLT3 inhibitor treatment.

(**A**) Volcano plots showing differentially translated proteins between FLT3 inhibitor (quizartinib, crenolanib, gilteritinib)-treated MV4-11 or MOLM-14 cells and DMSO-treated control cells (n=5). Log2 fold changes (FC) are plotted against FDR-corrected P values (DEqMS/limma-moderated two-sided t test). Red dots indicate significant increase in translation (FC > 0.25, P value < 0.05). (**B**) Ternary plots comparing log2 fold changes of newly translated proteins between quizartinib (Qui), crenolanib (Cre) and gilteritinib (Gil) treatments in MV4-11 (left) and MOLM-14 (right). For each protein and treatment, fold changes to DMSO were averaged across the replicates and plotted (n=5). (**C**) Translation of the human kinome. Fold changes in translation of kinases in MV4-11 cells (gilteritinib/DMSO, FDR < 0.1; n=5) of human kinases were Z score-normalized and are shown as a kinome dendrogram. Size and color of each kinase indicate its translation Z score. Empty dendrogram leaves indicate unmeasured kinases or kinases without changes in translation (FDR > 0.1). The distance along the branches between any two kinases is proportional to the sequence divergence of their catalytic domains; atypical kinases are grouped separately.

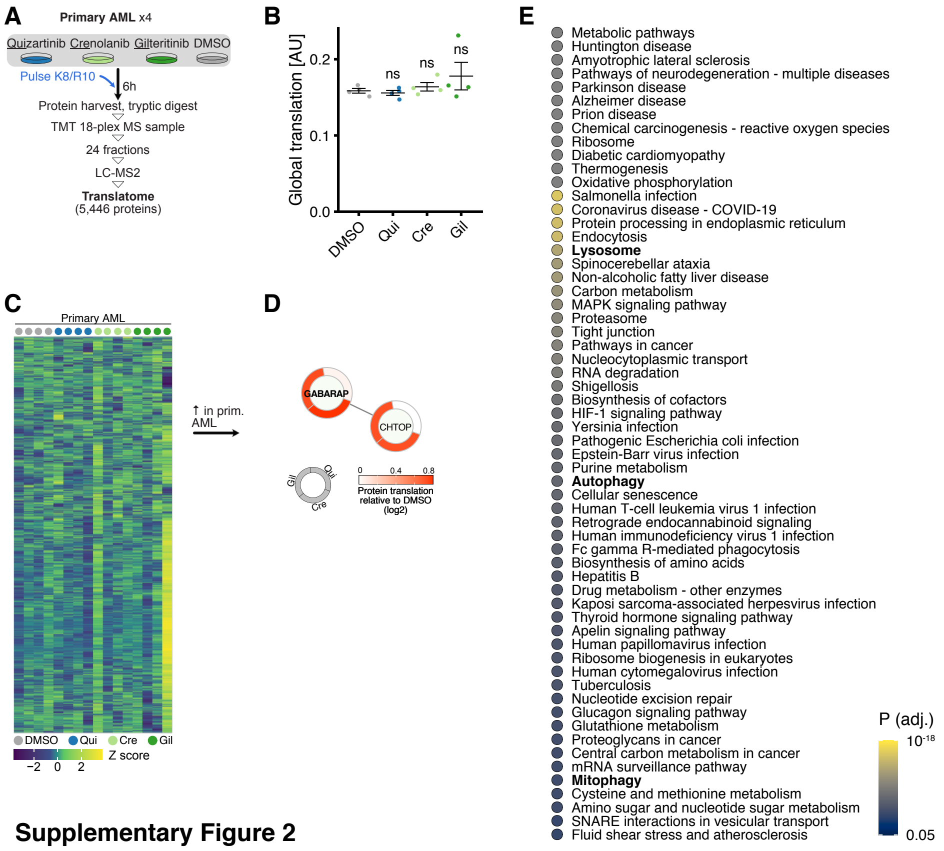

Supplementary Figure 2

Supplementary Fig. 2: Differential expression analysis of protein translation in primary AML cells following FLT3 inhibitor treatment.

(**A**) Experimental layout. Primary FLT3-ITD+ AML cells obtained by leukapheresis and ficoll gradient-density separation from a newly diagnosed, untreated AML patient were treated with 10 nM quizartinib (Qui), crenolanib (Cre), gilteritinib (Gil) or DMSO control for 6 hours (h) in Lys8- and Arg10-containing SILAC-heavy medium (n=4). (**B**) Summed abundances of all newly translated, SILAC-heavy labeled proteins following FLT3 inhibitors (FLT3i). Horizontal bars indicate mean, error bars show SEM; P values by two-sided paired t-test against DMSO (ns; not significant). (**C**) Heatmap showing row-scaled Z scores for all measured proteins across all conditions. (**D**) Network of proteins significantly upregulated following FLT3i. Nodes are proteins, circle segments around nodes indicate the average log2 FC for each FLT3 inhibitor. Autophagy-related proteins are manually highlighted by bold typeface. (**E**) KEGG pathway analysis on all proteins with increased translation upon FLT3i. The top 60 significant (P < 0.05, adjusted for multiple testing by g:SCS) annotations are shown.

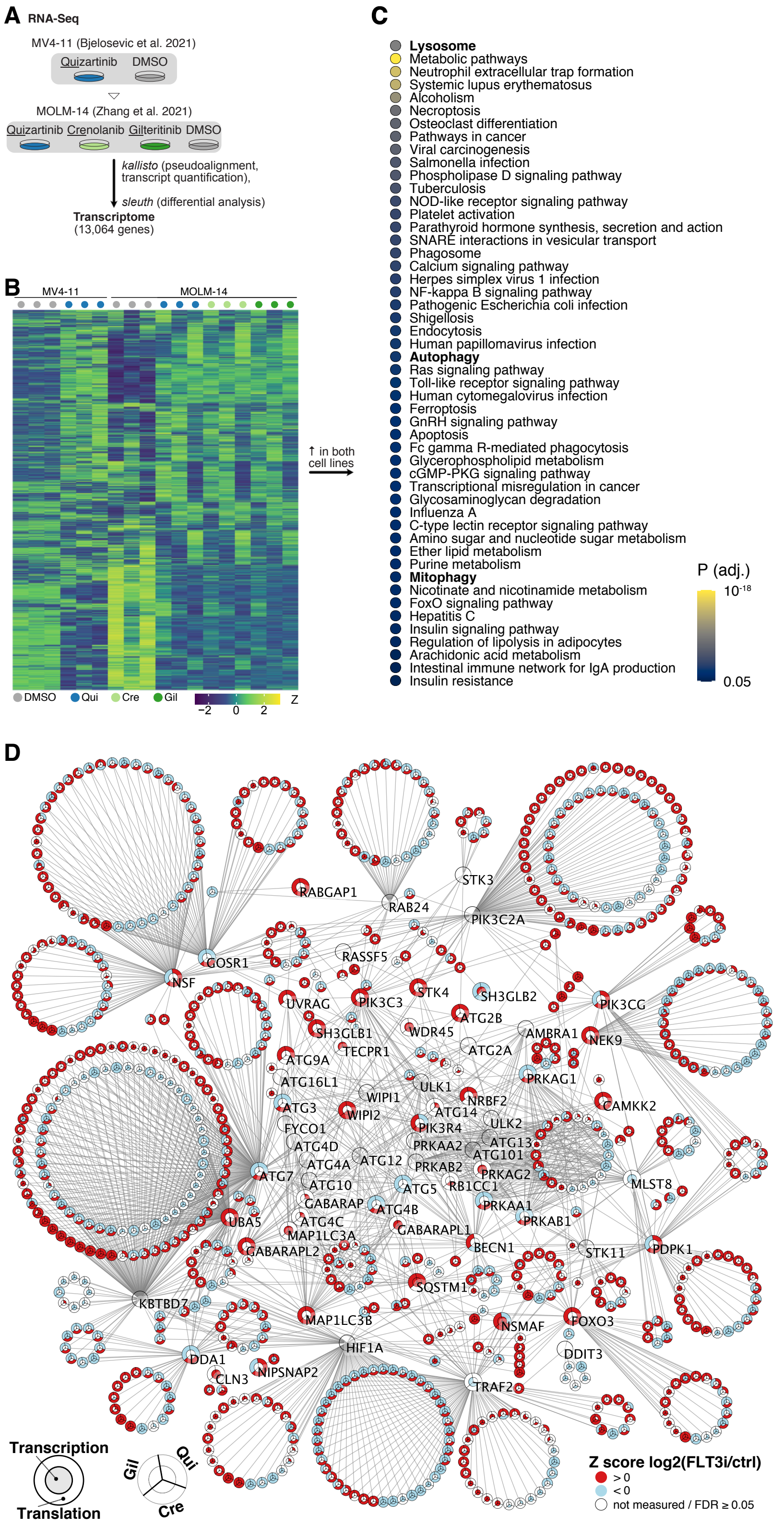

Supplementary Figure 3

Supplementary Fig. 3: Transcriptome sequencing identifies transcriptional induction of autophagy-related genes.

(**A**) Overview. Publicly available RNA-Seq datasets of MV4-11 cells treated with 5 nM quizartinib or vehicle control for 24 hours (14) and MOLM-14 cells treated with 2 nM quizartinib, crenolanib, gilteritinib or vehicle control for 24 hours (15) were re-analyzed. (**B**) Heat map showing row-scaled Z scores for all measured genes across all conditions. Z scores were calculated separately for the two datasets. (**C**) KEGG pathway analysis on genes upregulated in both cell lines following FLT3i (logFC > 0.25, q < 0.05). The top 50 significant (P < 0.05, adjusted for multiple testing by g:SCS) annotations are shown. (**D**) Translatome data (see Fig. 1) and transcriptome data for MOLM-14 cells mapped onto the network of human core autophagy proteins and their high-confidence (> 0.9) STRING interactors. Core autophagy proteins are labeled. Node fill color shows significant (FDR < 0.05), global Z-scored log2 FC in gene expression (inner circle) and protein translation (outer circle). Individual inner and our circle segments around nodes indicate changes upon quizartinib (right upper), crenolanib (lower) and gilteritinib (left upper) treatment.

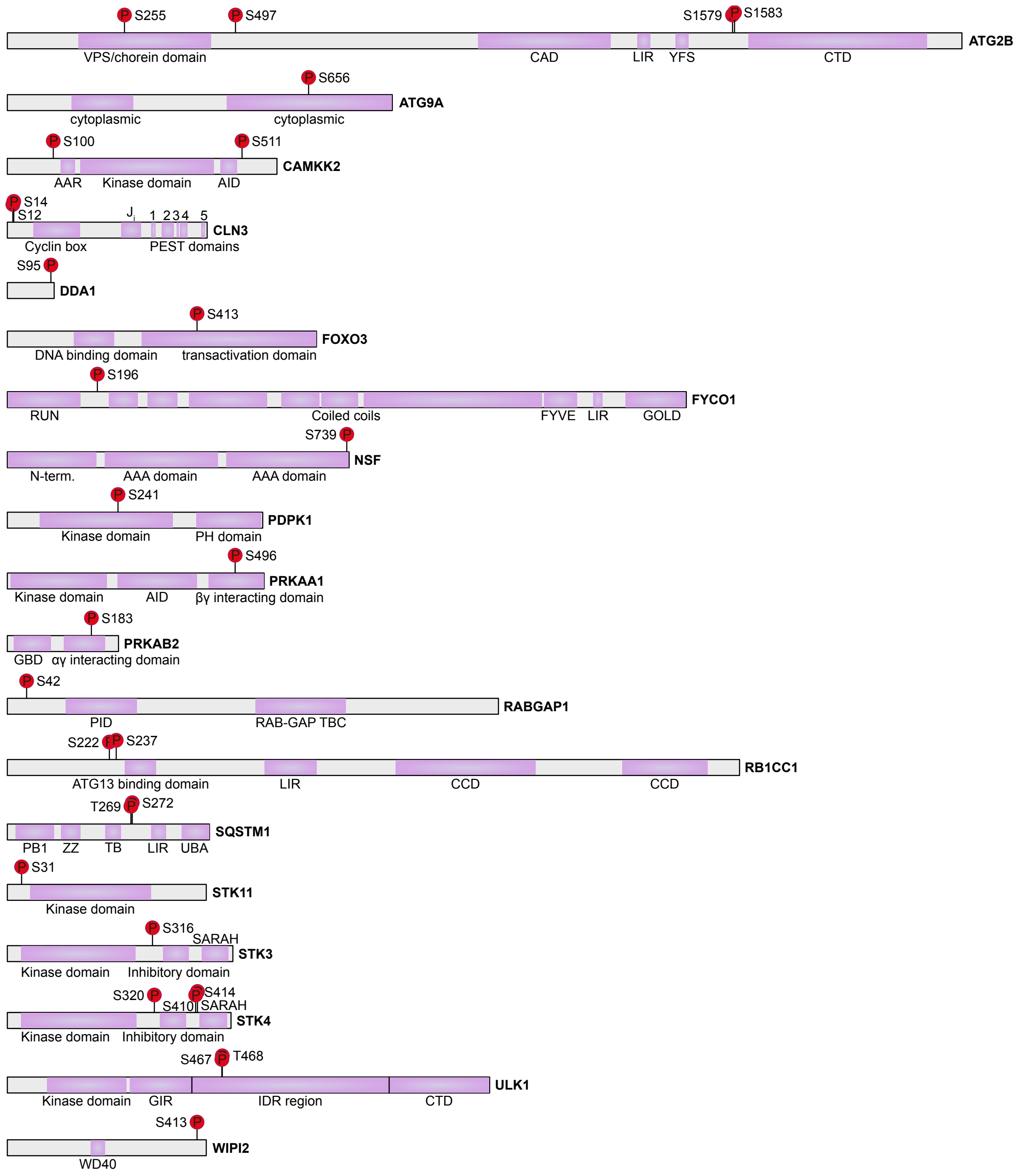

Supplementary Figure 4

Supplementary Fig. 4: Phosphosites on core autophagy proteins identified in both cell lines and primary AML cells.

Consensus phosphosites identified both in MV4-11 and primary AML cells (see Fig. 2) on core autophagy proteins. Sites are shown in relation to main protein domains. AAR, autonomous activity region; AID, autoinhibitory region; J_i, chaperone-regulatory domain; GBD, glykogen binding domain; PID, phosphotyrosine interaction domain; TBC, Tre-2/Bub2/Cdc16; CCD, coiled-coil domain; IDR, intrinsically disordered region; CTD, C-terminal domain.

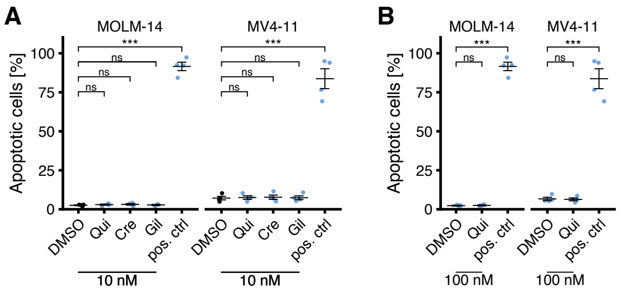

Supplementary Figure 5

Supplementary Fig. 5: Short-term FLT3 inhibitor treatment does not induce apoptosis or cell death.

(**A**) Flow cytometry measurements of MOLM-14 (n=4) and MV4-11 (n=4) cells treated with 10 nM quizartinib, crenolanib, gilteritinib or DMSO vehicle control for 6 hours and co-stained with Annexin-V (AnV) and 7AAD. Heat-boiled (95 °C for 60 seconds) cells served as a positive control. AnV+/7AAD-, AnV-/7AAD+ and AnV+/7AAD+ were regarded as apoptotic. Percentages are relative to all cellular events. Horizontal bar indicates mean, error bars show SEM; P values by two-sided paired t-test (*** P < 0.001, not significant (ns) P ≥ 0.05). (**B**) Same as (A), except that MOLM-14 (n=4) and MV4-11 (n=4) cells were treated with 100 nM quizartinib for 4 hours.

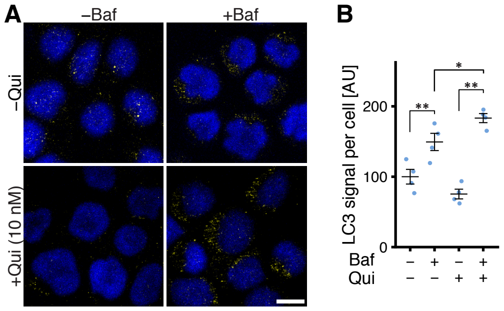

Supplementary Figure 6

Supplementary Fig. 6: Endogenous LC3 accumulation and quantification of autophagic flux induced by 10 nM quizartinib.

(**A**) Representative confocal microscopy images (maximum intensity projections) of MV4-11 cells immunostained against endogenous LC3A/B after 10 nM quizartinib (Qui) or DMSO vehicle for 4 h, ± concurrent inhibition of autophagosomal LC3 degradation by 100 nM Bafilomycin A1 (Baf) for 4 h. The scale is identical for all images. Scale bar (lower right) measures 10 μm. (**B**) Quantification of cytosolic LC3 signal (n=4). Values are scaled such that the overall mean of untreated samples is 100. Points

indicate means from individual experiments, horizontal bar denotes overall mean, error bars show SEM; P values by two-sided paired t-test (* P < 0.05, ** P < 0.01).

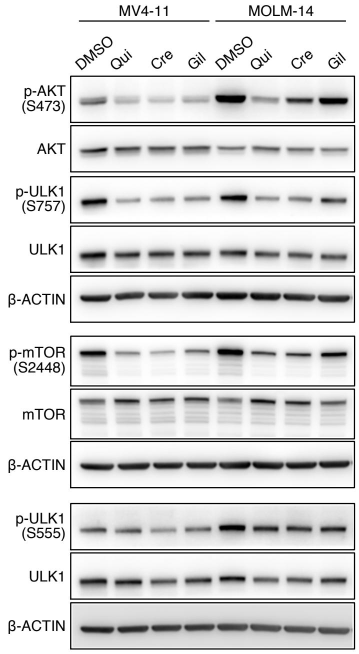

Supplementary Figure 7

Supplementary Fig. 7: Modulation of the FLT3/AKT/mTOR/ULK1 pathway in cell lines.

Immunoblots from Fig. 3G (lysates from MV4-11 and MOLM-14 cells treated with 10 nM quizartinib, crenolanib, gilteritinib or DMSO control for 4 h) rearranged according to physical layout on whole blots and shown with beta-actin loading controls (see also Source Figure Immunoblots for total protein membrane stains).

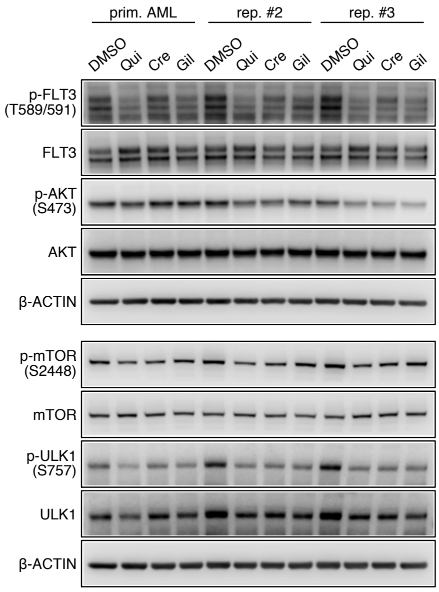

Supplementary Figure 8

Supplementary Fig. 8: Modulation of the FLT3/AKT/mTOR/ULK1 pathway in primary AML cells.

Immunoblots of lysates from primary FLT3-ITD+ AML cells treated with 10 nM quizartinib, crenolanib, gilteritinib or DMSO control for 24 h. Experiment was repeated with n=3 replicates (different sample aliquots) of one primary AML.

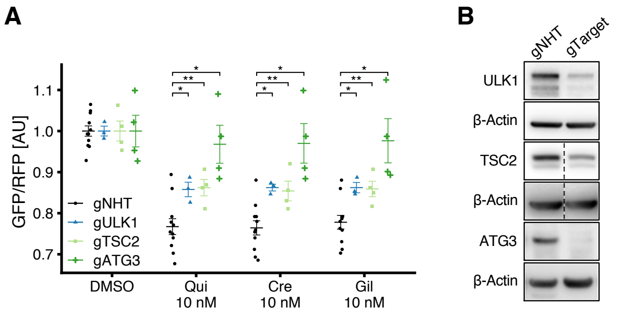

Supplementary Figure 9

Supplementary Fig. 9: CRISPR/Cas9-targeting of ULK1, TSC2 or ATG3 ablates autophagy induced by FLT3 inhibitors.

(**A**) Flow cytometry measurements of GFP-LC3B/RFP in MV4-11 Cas9 cells transduced with gRNA against ULK1, TSC2, ATG3 or non-human target (NHT) and treated with DMSO control or 10 nM quizartinib, crenolanib or gilteritinib for 4 h (gULK1, n=3; gTSC2, n=4; gATG3, n=4). Values are scaled such that the overall mean of each genotype's DMSO condition is 1. Horizontal bar indicates mean, error bars show SEM; P values by two-sided paired t-test (* $P < 0.05$, ** $P < 0.01$, *** $P < 0.001$). See also Fig. 3I (treatment with 100 nM FLT3i). (**B**) Immunoblots of CRISPR/Cas9-edited MV4-11 cells. Total levels of ULK1, TSC2, ATG3 and β-Actin in polyclonal MV4-11 cells transduced with SpCas9 and gRNA targeting ULK1, TSC2 or ATG3 or non-human target (NHT). For TSC2 and its loading control, bands from non-adjacent lanes from the same blot are shown.

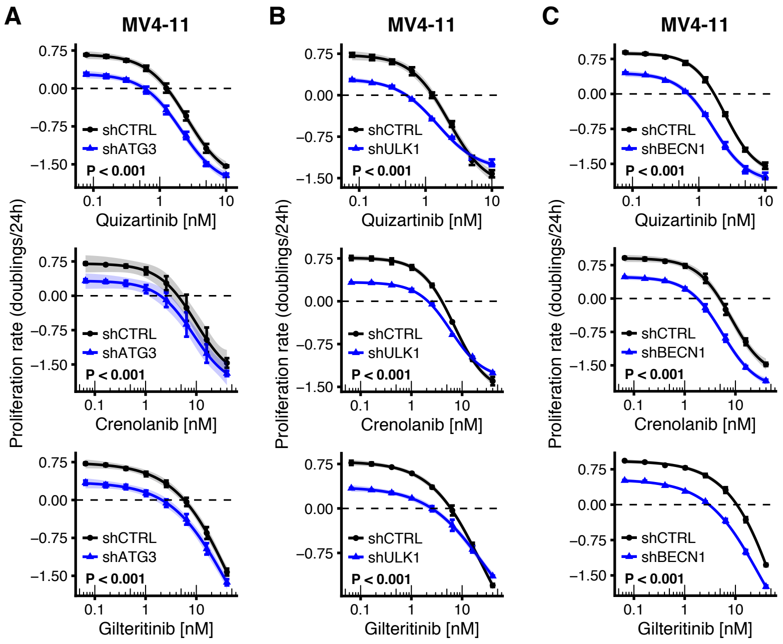

Supplementary Figure 10

Supplementary Fig. 10: Time-averaged proliferation rates of FLT3 inhibitor treated MV4-11 cells with genetic autophagy impairment.

(**A**) Time-averaged proliferation rates (cell number doublings/24 h) of MV4-11 cells transduced with shRNA against ATG3 (shATG3), (**B**) ULK1 (shULK1), (**C**) BECLIN1 (shBECN1) or nun-human target (shCTRL) and treated with varying concentrations of quizartinib, crenolanib or gilteritinib for 72 h (n=3). Cell numbers were measured every 24 h by flow cytometry. Dots indicate mean, error bars show SEM, shaded regions indicate 95% confidence interval of the four-parameter log-logistic dose-response models; P values by F test (*** $P < 0.001$).

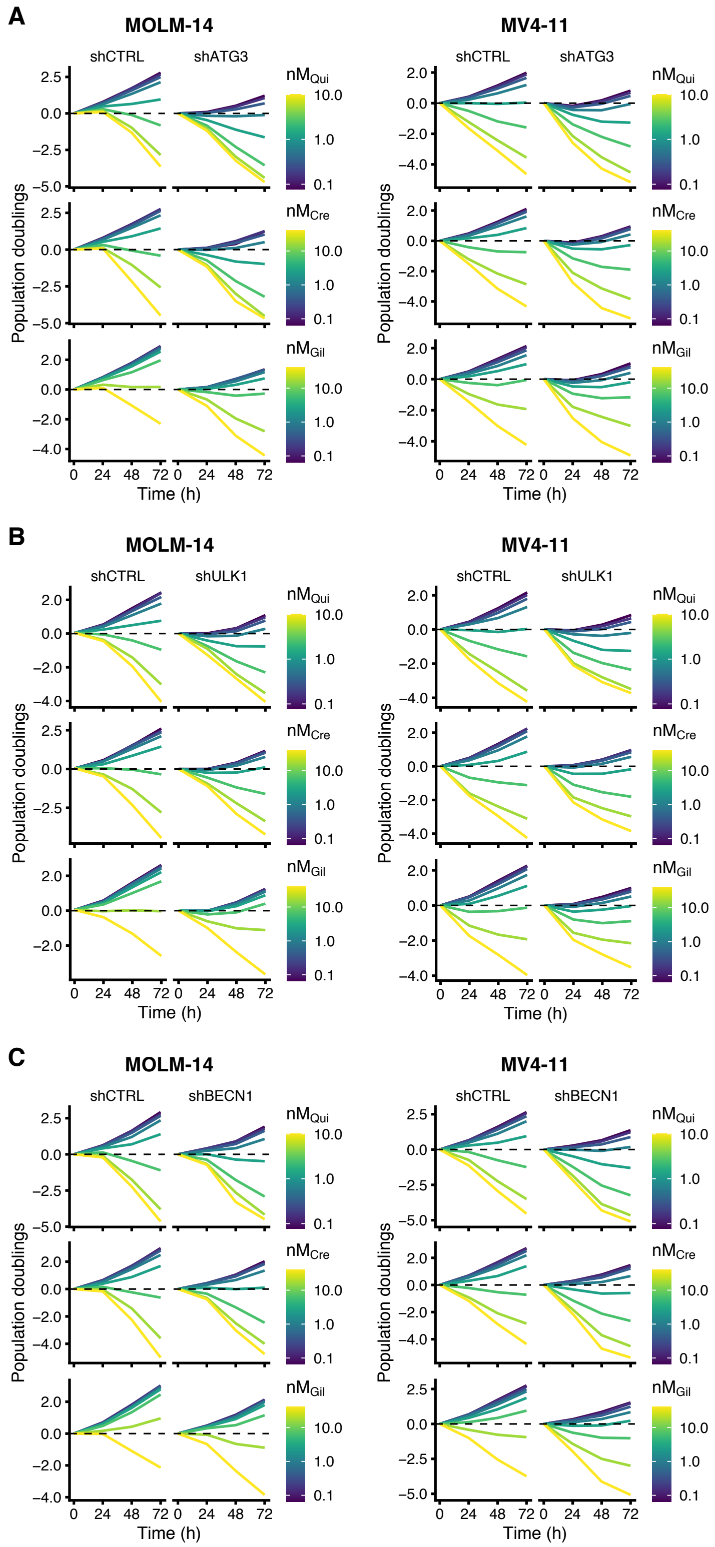

Supplementary Figure 11

Supplementary Fig. 11: Proliferation kinetics of FLT3 inhibitor-treated cells with genetic autophagy impairment.

(**A**) Mean cell number doublings of MOLM-14 (left) and MV4-11 cells (right) transduced with shRNA against non-human target (shCTRL) or ATG3 (shATG3) or (**B**) ULK1 (shULK1) or (**C**) BECLIN1

(shBECN1) and treated with varying concentrations of quizartinib, crenolanib or gilteritinib for 72 h (n=3). Profiles are color-coded by FLT3 inhibitor concentrations. Related to Fig. 4A-C and Supplementary Fig. 10.

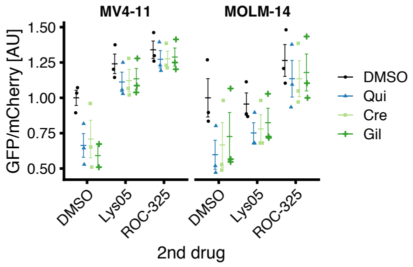

Supplementary Figure 12

Supplementary Fig. 12: Lysosomal autophagy inhibitors effectively impair autophagy induction by FLT3 inhibitors.

MV4-11 (n=3) and MOLM-14 cells (n=3) expressing GFP-LC3B-mCherry autophagy flux reporter were co-treated for 6h with either 10 nM quizartinib (Qui), crenolanib (Cre) or gilteritinib (Gil) or DMSO (1st drug) and either 5 µM ROC-325, Lys05 or DMSO (2nd drug). GFP/mCherry was measured by flow cytometry. Values are scaled by a global constant such that the overall mean of each cell line's DMSO+DMSO condition is 1. Horizontal bars indicate mean, error bars show SEM. AU, arbitrary units.

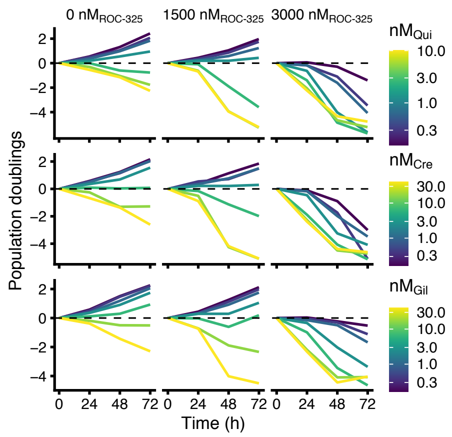

Supplementary Figure 13

Supplementary Fig. 13: Proliferation kinetics of cells co-treated with FLT3 inhibitors and autophagy inhibitor

Mean cell number doublings of MV4-11 cells co-treated with ROC-325 and quizartinib (Qui), crenolanib (Cre) or gilteritinib (Gil) and measured every 24 h by flow cytometry. Profiles are color-coded by FLT3i concentrations and shown for selected concentrations of ROC-325 (n=3; related to Fig. 4D).

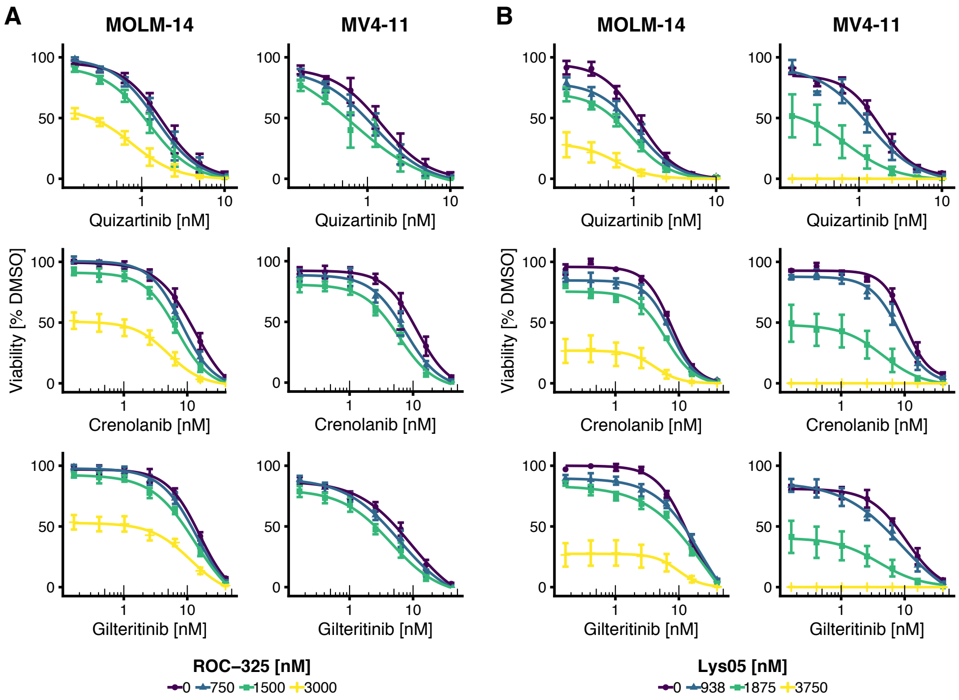

Supplementary Figure 14

Supplementary Fig. 14: Viability end-point assays on FLT3-ITD+ cell lines co-treated with FLT3 inhibitors and autophagy inhibitors.

(**A**) Dose-response curves showing cell viabilities (relative to DMSO vehicle-treated cells) of MOLM-14 and MV4-11 cells co-treated with FLT3 inhibitors (FLT3i) quizartinib, crenolanib or gilteritinib and ROC-325 for 72 h (n=4). Profiles are color-coded by autophagy inhibitor concentration. Dots indicate mean, error bars show SEM. Related to Fig. 4G (quantification of synergism). (**B**) Experiment as in (A), except that cells were co-treated with lysosomal autophagy inhibitor Lys05 instead of ROC-325 (n=4). Related to Fig. 4H (quantification of synergism).

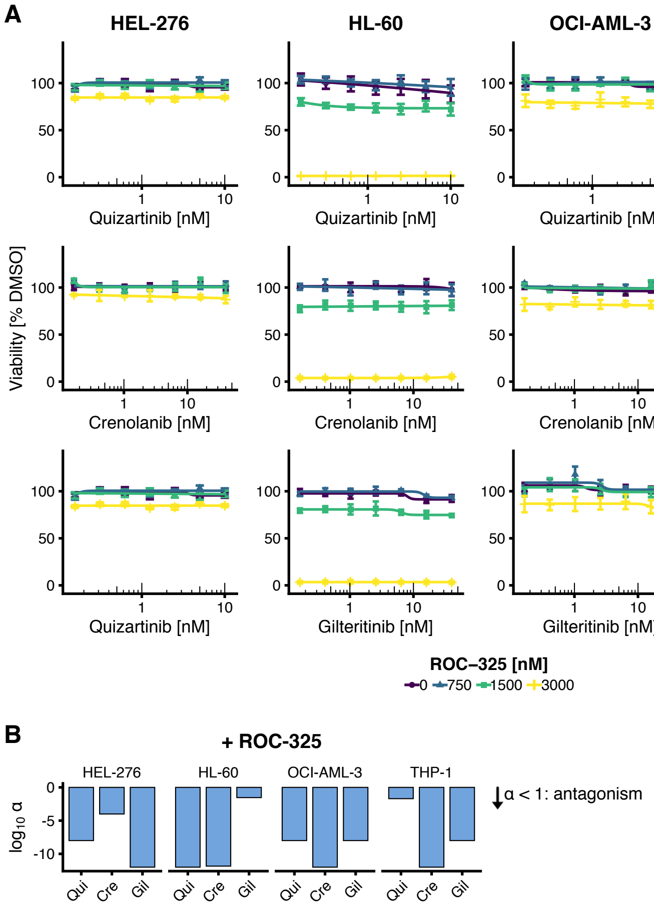

Supplementary Figure 15

Supplementary Fig. 15: Viability end-point assays on FLT3 wildtype cell lines co-treated with FLT3 inhibitors and autophagy inhibitor.

(**A**) Dose-response curves showing cell viabilities (relative to DMSO vehicle-treated cells) of HEL-276, HL-60, OCI-AML-3 and THP-1 cells (all FLT3 wildtype) co-treated with FLT3 inhibitors (FLT3i) quizartinib, crenolanib or gilteritinib and ROC-325 for 72 h (n=4 each). Profiles are color-coded by autophagy inhibitor concentration. Dots indicate mean, error bars show SEM. (**B**) Quantification of synergistic/antagonistic potency α in endpoint viability assays shown in (A) by MuSyC. α < 1 indicates antagonism, α > 1 indicates synergism.

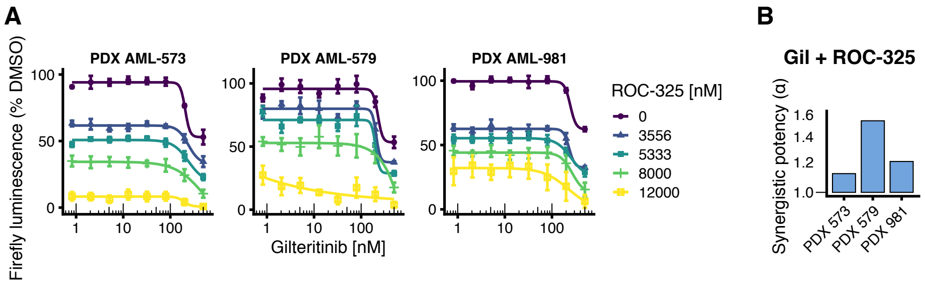

Supplementary Figure 16

Supplementary Fig. 16: Viability of PDX cells co-treated with FLT3 inhibitor and autophagy inhibitor.

Dose-response curves showing luminescence quantification (relative to DMSO vehicle) of FLT3-ITD+ AML patient-derived xenograft (PDX) cells stably expressing firefly luciferase after treatment with gilteritinib, ROC-325, or the combination thereof for 72 h. Dots indicate mean, error bars show SEM (AML-573, n=3; AML-579, n=4; AML-981, n=4).

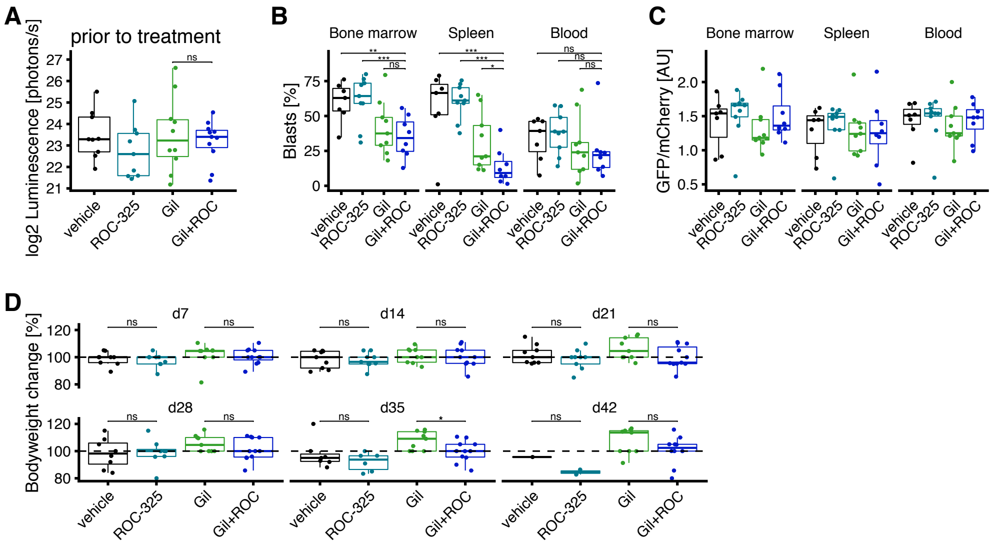

Supplementary Figure 17

Supplementary Fig. 17: Engraftment, organ infiltration, *ex vivo* autophagy flux measurements and body weight measurements of NSG mice xenografted with MV4-11 cells

(**A**) Quantification of bioluminescence for individual mice two weeks after xenotransplantation prior to treatment initiation with either vehicle (n=9), ROC-325 (n=9), gilteritinib (n=10), or gilteritinib + ROC-325 (n=11). P values by two-sided unpaired t-test (ns, not significant). (**B**) Leukemic infiltration of murine bone marrow, spleen and blood after treatment at death. Blast percentages of all isolated cells as determined by flow cytometry are displayed. P values by beta regression (ns, not significant; *, $P < 0.05$; **, $P < 0.01$, *** $P < 0.001$). Individual animals with organ analyses available are shown (vehicle, n=7; ROC-325, n=9, gilteritinib, n=9; gilteritinib + ROC-325, n=8). (**C**) Flow cytometry measurements of GFP-LC3B/mCherry in isolated blasts (see B). AU, arbitrary units. (**D**) Changes in bodyweight relative to the day when daily drug treatment was initiated. P values by two-sided t test (ns, not significant; *, $P < 0.05$).

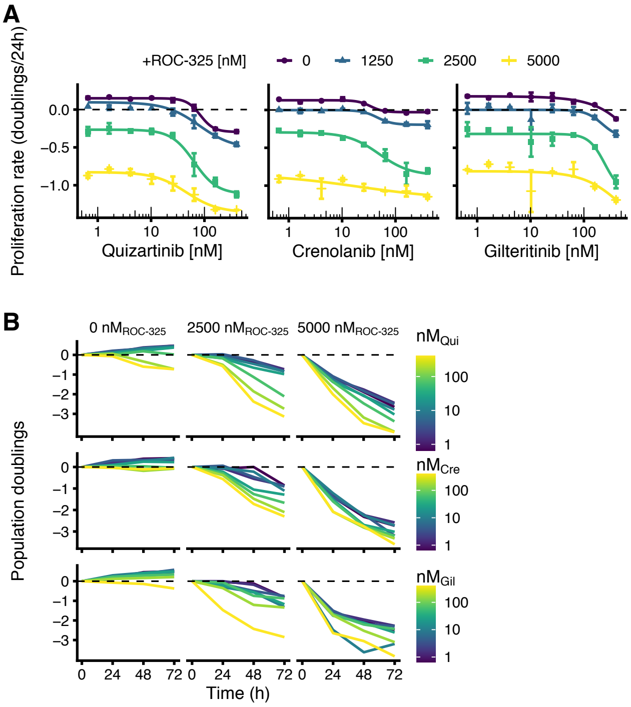

Supplementary Figure 18

Supplementary Fig. 18: Proliferation kinetics of primary human AML cells co-treated with FLT3i and autophagy inhibitor.

(**A**) Dose-response curves (four-parameter log-logistic function) showing time-averaged proliferation rates (cell number doublings/24 h) of primary cells from individual FLT3-ITD+ AML patient #1 (leukapheresate) co-treated with FLT3 inhibitors and autophagy inhibitor ROC-325 at varying concentrations for 72 h (n=2 repeated experiments on separate sample aliquots from the same patient). Cell numbers were measured every 24 h by flow cytometry. Dots indicate mean, error bars show SEM. (**B**) Mean cell number doublings of individual AML sample #1 (leukapheresate) co-treated with FLT3i and lysosomal autophagy inhibitor ROC-325 at varying concentrations and measured by flow cytometry every 24 h (n=2 repeated experiments on separate sample aliquots of the same patient).

Source data for Figure 3, Supplementary Figure 7, Supplementary Figure 8, Supplementary Figure 9

a Source data for **Fig. 3G** and **Supplementary Figure 7**. Unprocessed immunoblots (full-length membrane cuts)

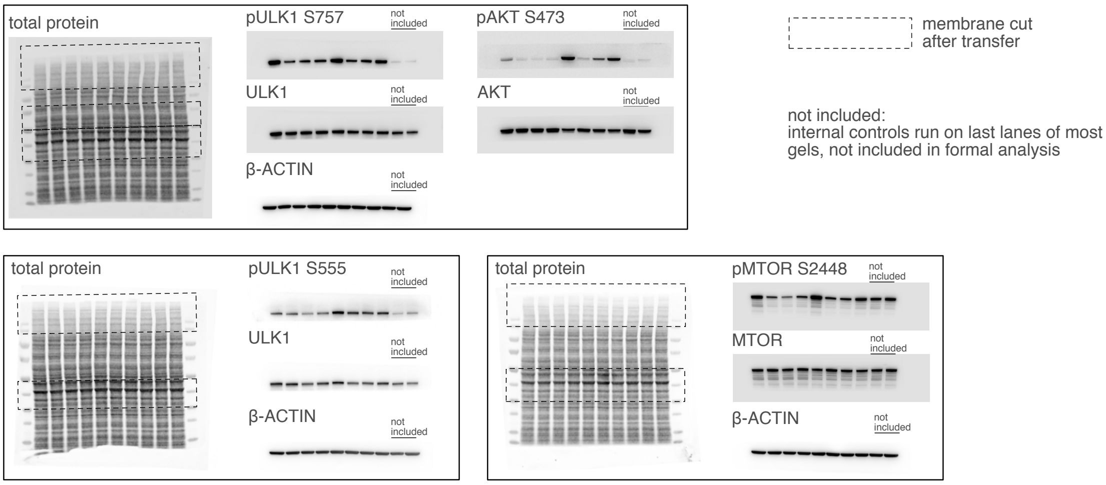

b Source data for **Fig. 3H**. Unprocessed immunoblots (additional replicates)

Replicate

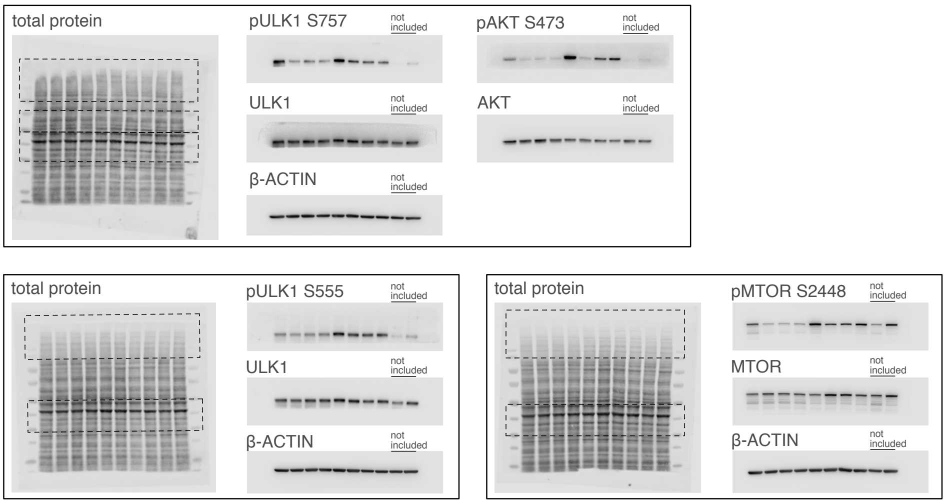

Source data for Figure 3, Supplementary Figure 7, Supplementary Figure 8, Supplementary Figure 9

Replicate

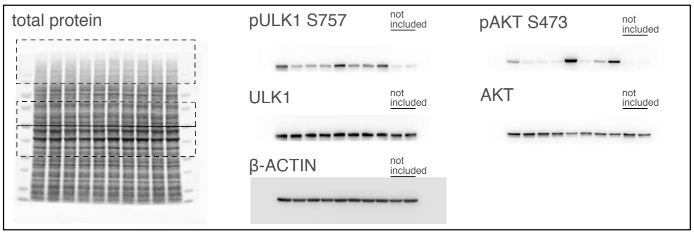

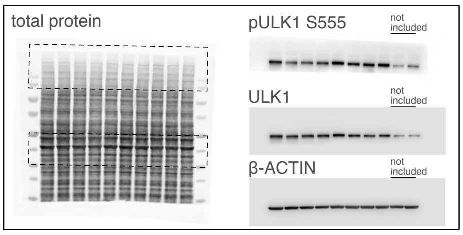

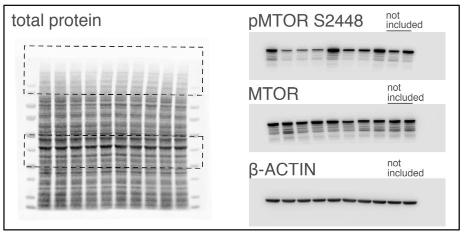

Replicate

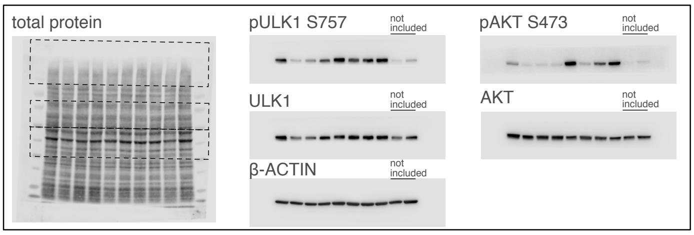

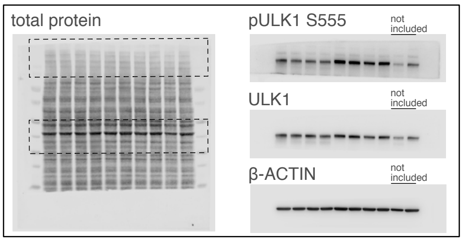

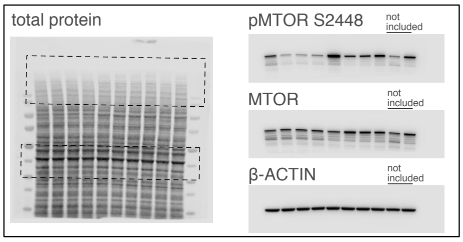

Replicate

(MV4-11 not included due to technical error)

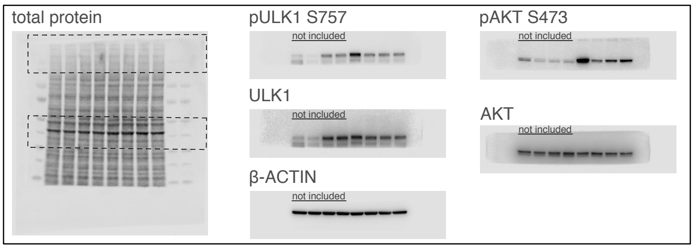

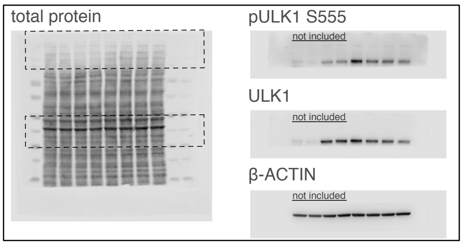

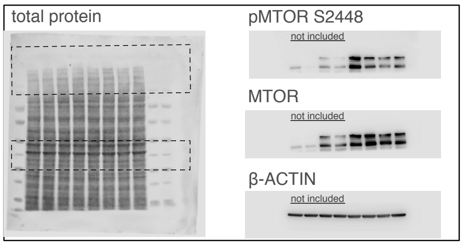

Source data for Figure 3, Supplementary Figure 7, Supplementary Figure 8, Supplementary Figure 9

c Source data for **Supplementary Figure 8**. Unprocessed immunoblots (full-length membrane cuts)

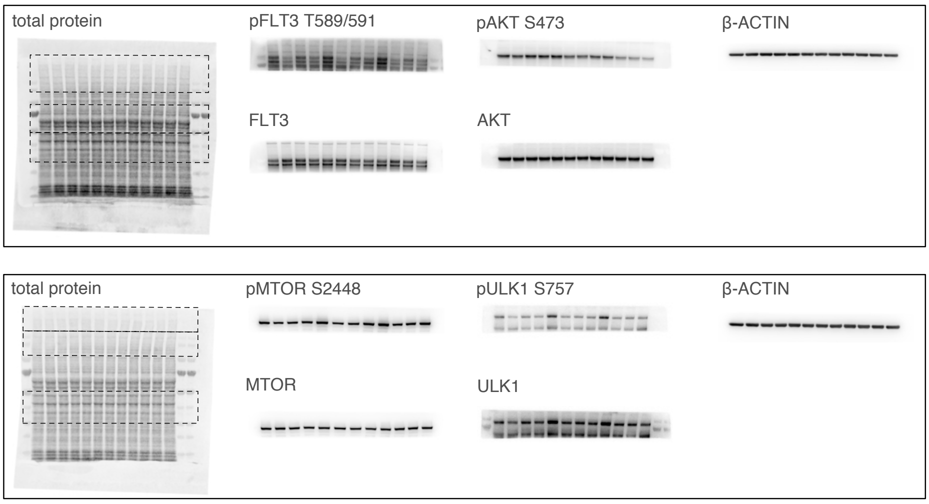

d Source data for **Supplementary Fig. 9**. Unprocessed immunoblots (full-length membrane cuts)

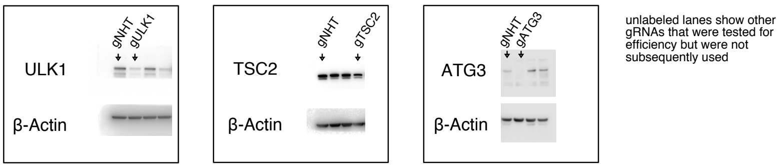

Supplementary Source Figure Immunoblots: All unprocessed immunoblots shown in the main manuscript, with all replicates used for quantification (PDF).

Supplementary Tables

Supplementary Table 1: Processed translation proteomics data (XLSX)

Supplementary Table 2: Processed phosphoproteomics data (XLSX)

		All patients (n = 11)
Gender	female	7 (64 %)
	male	4 (36 %)
Age, years		60 (20—81)
Blood parameters at diagnosis	Bone marrow blasts, %	73 (40—88)
	Peripheral blood blasts, %	74 (4—92)
	White blood cell count, cells/nl	85 (2—235)
	Platelet count, cells/nl	52 (20—152)
	LDH, U/L	810 (249—1402)
Genetics	Normal karyotype	8 (73 %)
	Aberrant karyotype, < 3 aberrations	3 (27 %)
	FLT3-ITD	11 (100 %)
	NPM1c	5 (45 %)
ELN2017 risk group	Favorable	1 (9 %)
	Intermediate	7 (64 %)
	Adverse	3 (27 %)
Sample	Bone marrow aspiration	10 (91 %)
	Leukapheresis	1 (9 %)

Supplementary Table 3: AML patient and sample characteristics

Characteristics of AML patients/samples used in the ex vivo experiments. Data are n (%) or median with range. LDH, lactate dehydrogenase; ITD, internal tandem duplication, ELN2017, revised 2017 European LeukemiaNet risk classification.

Supplementary References

1. Klann K, Tascher G, Münch C. Functional Translatome Proteomics Reveal Converging and Dose-Dependent Regulation by mTORC1 and eIF2α. Mol Cell. 2020;77:913-925.e4.
2. Plubell DL, Wilmarth PA, Zhao Y, Fenton AM, Minnier J, Reddy AP et al. Extended Multiplexing of Tandem Mass Tags (TMT) Labeling Reveals Age and High Fat Diet Specific Proteome Changes in Mouse Epididymal Adipose Tissue. Mol Cell Proteomics. 2017;16:873-890.
3. Bray NL, Pimentel H, Melsted P, Pachter L. Near-optimal probabilistic RNA-seq quantification. Nat Biotechnol. 2016;34:525-527.
4. Pimentel H, Bray NL, Puente S, Melsted P, Pachter L. Differential analysis of RNA-seq incorporating quantification uncertainty. Nat Methods. 2017;14:687-690.
5. Nguyen TD, Shaid S, Vakhrusheva O, Koschade SE, Klann K, Thölken M et al. Loss of the selective autophagy receptor p62 impairs murine myeloid leukemia progression and mitophagy. Blood. 2019;133:168-179.
6. Schneider CA, Rasband WS, Eliceiri KW. NIH Image to ImageJ: 25 years of image analysis. Nat Methods. 2012;9:671-675.
7. Linkert M, Rueden CT, Allan C, Burel JM, Moore W, Patterson A et al. Metadata matters: access to image data in the real world. J Cell Biol. 2010;189:777-782.
8. Harris LA, Frick PL, Garbett SP, Hardeman KN, Paudel BB, Lopez CF et al. An unbiased metric of antiproliferative drug effect in vitro. Nat Methods. 2016;13:497-500.
9. Vick B, Rothenberg M, Sandhöfer N, Carlet M, Finkenzeller C, Krupka C et al. An advanced preclinical mouse model for acute myeloid leukemia using patients' cells of various genetic subgroups and in vivo bioluminescence imaging. PLoS One. 2015;10:e0120925.
10. Luo S, Wehr NB, Levine RL. Quantitation of protein on gels and blots by infrared fluorescence of Coomassie blue and Fast Green. Anal Biochem. 2006;350:233-238.
11. Shannon P, Markiel A, Ozier O, Baliga NS, Wang JT, Ramage D et al. Cytoscape: a software environment for integrated models of biomolecular interaction networks. Genome research. 2003;13:2498-2504.
12. Raudvere U, Kolberg L, Kuzmin I, Arak T, Adler P, Peterson H et al. g:Profiler: a web server for functional enrichment analysis and conversions of gene lists (2019 update). Nucleic Acids Res. 2019;47:W191-W198.

13. Zhang B, Horvath S. A general framework for weighted gene co-expression network analysis. Stat Appl Genet Mol Biol. 2005;4:Article17.
14. Bjelosevic S, Gruber E, Newbold A, Shembrey C, Devlin JR, Hogg SJ et al. Serine biosynthesis is a metabolic vulnerability in FLT3-ITD-driven acute myeloid leukaemia. Cancer Discov. 2021
15. Zhang Y, Newsom KJ, Zhang M, Kelley JS, Starostik P. GATM-Mediated Creatine Biosynthesis Enables Maintenance of FLT3-ITD-Mutant Acute Myeloid Leukemia. Mol Cancer Res. 2021
